# A novel panel of yeast assays for the assessment of thiamin and its biosynthetic intermediates in plant tissues

**DOI:** 10.1111/nph.17974

**Published:** 2022-02-08

**Authors:** Simon Strobbe, Jana Verstraete, Teresa B. Fitzpatrick, Maria Faustino, Tiago F. Lourenço, M. Margarida Oliveira, Christophe Stove, Dominique Van Der Straeten

**Affiliations:** ^1^ Laboratory of Functional Plant Biology Department of Biology Ghent University K.L. Ledeganckstraat 35 B‐9000 Gent Belgium; ^2^ Laboratory of Toxicology Department of Bioanalysis Ghent University Ottergemsesteenweg 460 B‐9000 Gent Belgium; ^3^ Vitamins and Environmental Stress Responses in Plants Department of Botany and Plant Biology University of Geneva Quai E. Ansermet 30 1211 Geneva Switzerland; ^4^ Instituto de Tecnologia Química e Biológica António Xavier Universidade NOVA de Lisboa Plant Functional Genomics – GPlantS Lab Av. da República 2780‐157 Oeiras Portugal

**Keywords:** biofortification, metabolic engineering, microbiological assays, nutritional improvement, *Saccharomyces cerevisiae*, turbidimetry, vitamin quantification

## Abstract

- Thiamin (or thiamine), known as vitamin B1, represents an indispensable component of human diets, being pivotal in energy metabolism. Thiamin research depends on adequate vitamin quantification in plant tissues. A recently developed quantitative liquid chromatography–tandem mass spectrometry (LC–MS/MS) method is able to assess the level of thiamin, its phosphorylated entities and its biosynthetic intermediates in the model plant *Arabidopsis thaliana*, as well as in rice. However, their implementation requires expensive equipment and substantial technical expertise. Microbiological assays can be useful in deter‐mining metabolite levels in plant material and provide an affordable alternative to MS‐based analysis.
- Here, we evaluate, by comparison to the LC–MS/MS reference method, the potential of a carefully chosen panel of yeast assays to estimate levels of total vitamin B1, as well as its biosynthetic intermediates pyrimidine and thiazole in Arabidopsis samples.
- The examined panel of *Saccharomyces cerevisiae* mutants was, when implemented in microbiological assays, capable of correctly assigning a series of wild‐type and thiamin biofortified Arabidopsis plant samples.
- The assays provide a readily applicable method allowing rapid screening of vitamin B1 (and its biosynthetic intermediates) content in plant material, which is particularly useful in metabolic engineering approaches and in germplasm screening across or within species.

## Introduction

Thiamin is an essential micronutrient and was the first compound characterized within the group of B vitamins, hence vitamin B1 (vitB1) (Lonsdale, 2006; Fitzpatrick & Chapman, 2020). Although only minute amounts are present in the human body, the active derivative of thiamin, thiamin pyrophosphate (TPP), serves as a cofactor of several enzyme complexes (transketolase, pyruvate dehydrogenase, α‐ketoglutarate dehydrogenase and branched‐chain ketoacid dehydrogenase) involved in energy metabolism. Despite having lost the ability to synthesize thiamin *de novo*, the human body does have the metabolic capacity to interconvert the different (non‐)phosphorylated entities of vitB1 (thiamin, thiamin monophosphate (TMP) and TPP), referred to as B1 vitamers (Rao & Butterworth, 1995; Nosaka *et al*., 2001). Given that the body only stores limited amounts of thiamin, humans are relying on a continuous dietary intake of vitB1 via consumption of thiamin‐rich foods. The recommended daily allowance (RDA) for thiamin is 1.2 mg d^−1^ for men and 1.1 mg d^−1^ for women, with increased requirements during pregnancy (1.4 mg d^−1^) and lactation (1.5 mg d^−1^) (Whitfield *et al*., 2018). Severe thiamin deficiency can result in a pathological disorder called Beriberi, which can be divided in wet and dry Beriberi depending on whether it manifests in the cardiovascular or nervous system, respectively (DiNicolantonio *et al*., 2018; Wilson, 2020).

Biofortification, which involves the enhancement of micronutrient levels in crops, via conventional breeding or genetic engineering, has been stated as a cost‐effective strategy to increase the natural thiamin content in food crops (Goyer, 2017; Strobbe & Van Der Straeten, 2018; Fitzpatrick & Chapman, 2020). Both metabolic engineering strategies as well as conventional breeding endeavors can be considered powerful and sustainable methodologies to enhance thiamin intake of populations suffering from deficiency, as these can be continually deployed requiring a one‐time investment only (Bouis & Saltzman, 2017; Garg *et al*., 2018; Van Der Straeten *et al*., 2020).

However, the set‐up and monitoring of both approaches relies on adequate vitamin quantification in plant tissues. Indeed, conventional breeding requires assessment of the vitamin content in sexually compatible germplasm to allow the selection of interesting varieties to be included in the breeding program (Saltzman *et al*., 2013; Jiang *et al*., 2021). Similarly, metabolic engineering strategies rely on accurate vitamin measurement to select the most successful strategies as well as transgenic events (Verstraete *et al*., 2020, 2021). Moreover, quantification of biosynthetic intermediates is highly recommended, particularly in the assessment of metabolic engineering approaches (Farre *et al*., 2014). The reasoning behind this is two‐fold. First, assessment of intermediate accumulation can provide information to identify metabolic hurdles, which could be obstructing satisfactory biosynthetic flux towards the end product (Schaub *et al*., 2005; Blancquaert *et al*., 2013; Pourcel *et al*., 2013; Strobbe *et al*., 2021b). Second, high accumulation of intermediates, occurring as an undesired side effect of the metabolic engineering strategy, should be monitored and limited (Schaub *et al*., 2005; Blancquaert *et al*., 2013; Pourcel *et al*., 2013; Strobbe *et al*., 2021b). Metabolic engineering also requires in‐depth genetic knowledge on the biosynthesis of the desired metabolite, thiamin in this case (Goyer, 2017; Strobbe & Van Der Straeten, 2018; Fitzpatrick & Chapman, 2020), which in turn relies on adequate quantification of both the end product and its biosynthetic intermediates.

**Fig. 1:**
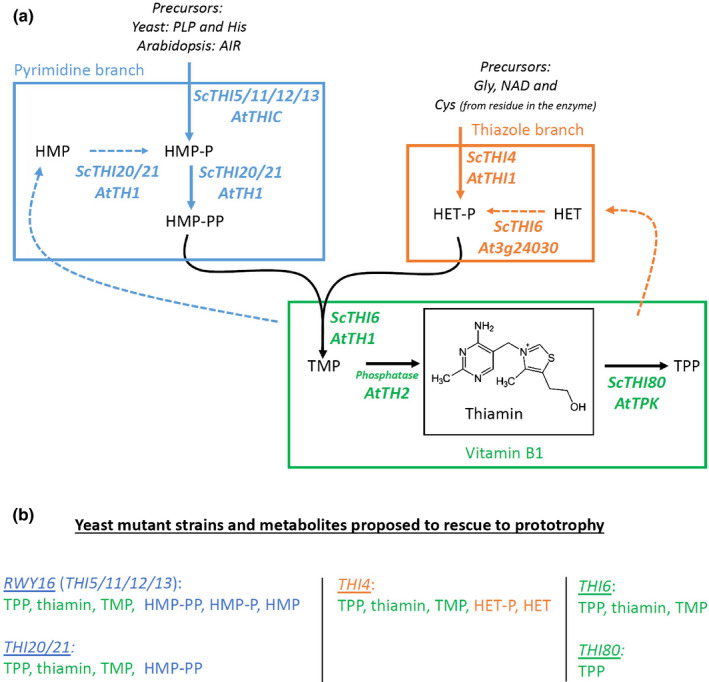
Thiamin metabolism in yeast and Arabidopsis and implications for rescuing yeast biosynthesis mutant lines. (a) Thiamin biosynthesis in *Saccharomyces cerevisiae* (*Sc*) and *Arabidopsis thaliana* (*At*). Full arrows indicate the thiamin biosynthesis *de novo* steps, dashed arrows indicate breakdown and enzymatic salvage of HMP and HET. The yeast (Sc) and Arabidopsis (At) genes, coding for the enzymes catalyzing the specific enzymatic step, are indicated. *AtTH1* (Ajjawi *et al*., 2007b) codes for a multifunctional enzyme executing steps catalyzed by the yeast enzymes coded by *ScTHI20*, *ScTHI21* and *ScTHI6*. Note that thiamin biosynthesis enzymes involved in the pyrimidine branch utilize different precursors in Arabidopsis as compared to yeast. The metabolites able to alleviate thiamin deficiency in human metabolism, therefore B1 vitamers, are indicated within the green square. The pyrimidine and thiazole branches of vitamin B1 (vitB1) biosynthesis are indicated within the blue and orange squares, respectively. (b) Five different *S. cerevisiae* mutant lines are listed and the different metabolites proposed to rescue each strain, based on the genetic and biosynthetic knowledge, are summarized. The list of metabolites is based on their occurrence in the thiamin biosynthesis pathway (metabolites downstream and/or able to bypass the mutated enzyme are proposed to rescue the mutant strain). The list of rescuing metabolites is deduced from the current knowledge of yeast thiamin metabolism, and assumes that (phosphorylated) metabolites can be adequately taken up by yeast cells. Supply of one (or multiple) of the listed metabolites is hypothesized to suffice to restore growth. Abbreviations: PLP, pyridoxal‐5′‐phosphate; His, histidine; AIR, aminoimidazole ribonucleotide; Gly, glycine; Cys, cysteine; NAD, nicotinamide adenine dinucleotide; HMP‐PP, 4‐amino‐2‐methyl‐5‐hydroxymethylpyrimidine ((pyro)phosphate); HET‐P, 4‐methyl‐5‐β‐hydroxyethylthiazole (phosphate); TMP, thiamin monophosphate; TPP, thiamin pyrophosphate.

Quantification of thiamin and its phosphate esters can be achieved via high‐performance liquid chromatography (HPLC) (Moulin *et al*., 2013; Martinis *et al*., 2016; Noordally *et al*., 2020). Recently, we described a quantitative liquid chromatography–tandem mass spectrometry (LC‐MS/MS) method for the determination of thiamin, its biosynthetic intermediates (4‐amino‐2‐methyl‐5‐hydroxymethylpyrimidine (HMP) and 4‐methyl‐5‐(2‐hydroxyethyl) thiazole (HET)) and phosphate derivatives (TMP and TPP) in *Arabidopsis thaliana* (Verstraete *et al*., 2020) and in rice (*Oryza sativa*) (Verstraete *et al*., 2021). As *A. thaliana* serves as a reference plant for plant genetics, the plant material is perfectly suited to perform preliminary genetic research to gain new insights into (regulation of) thiamin biosynthesis (Dong *et al*., 2015; Noordally *et al*., 2020; Strobbe *et al*., 2021b). Although the aforementioned HPLC and LC–MS/MS methods are highly sensitive, specific and precise, an extensive sample preparation procedure is required (Martinis *et al*., 2016; Verstraete *et al*., 2020). Moreover, these methods involve a substantial investment as specialized equipment is used, and implies substantial technical expertise. Alternatively, microbiological assays have been traditionally used to monitor the vitamin contents of foods (Schultz *et al*., 1942; Niven & Smiley, 1943; Edwards *et al*., 2017), in assessing crop natural variation (Mangel *et al*., 2017), and in determination of vitamin content (Tambasco‐Studart *et al*., 2005; Titiz *et al*., 2006; Raschke *et al*., 2007, 2011; Coquille *et al*., 2012; Li *et al*., 2015; Mangel *et al*., 2019). Affordability as well as simplicity are two important assets of microbiological assays. However, main disadvantages are their commonly low specificity and sensitivity. Turbidimetric assays for thiamin determination have been developed using a variety of organisms (for a historical overview, we refer to Edwards *et al*., 2017). Utilization of baker's yeast, *Saccharomyces cerevisiae*, offers the advantage to exploit the expanding library of mutant backgrounds, as well as the use of standard laboratory procedures to allow straightforward cultivation. More recently, such a yeast assay has been described for the determination of total vitB1 in Arabidopsis and cassava (*Manihot esculenta*) plant materials (Raschke *et al*., 2007; Mangel *et al*., 2017). However, no additional information about the biosynthetic intermediates (HMP and HET) was deduced using this assay. An overview of the *de novo* thiamin biosynthesis pathway in yeast, with indication of the orthologous Arabidopsis genes, is shown in Fig. 1(a). The aforementioned yeast assay uses a thiamin auxotrophic thiazole biosynthesis mutant (*thi4*) (Raschke *et al*., 2007; Chatterjee *et al*., 2011) (Fig. 1). The latter is proposed to be rescued by a range of metabolites, not only including the thiamin vitamers, but also thiazole biosynthetic intermediates (both HET and HET‐P; Fig. 1b) (Nosaka, 2006). Indeed, based on the knowledge of thiamin biosynthesis in *S. cerevisiae* (Nosaka, 2006; Fig. 1), supplementation of both HET and HET‐P (as well as the different B1 vitamers) should be able to restore prototrophy in the *thi4* strain, as these metabolites can be converted to B1 vitamers without the need for THI4 activity. Therefore, utilization of this strain in a microbiological assay might not be able to distinguish between these thiamin‐related metabolites, thus hampering the assessment of lines harboring high thiazole (e.g. resulting from metabolic engineering interventions).

The use of a panel of different mutant yeast strains, auxotrophic for different metabolites (Fig. 1b), may represent a novel way to gain insight into the metabolite profile of plant samples. Therefore, we examined the potential of using parallel yeast (*S. cerevisiae*) assays to assess the content of vitB1 (sum of thiamin, TMP and TPP) as well as its biosynthetic intermediates (pyrimidine and thiazole derivates; representing the totality of HMP and HET with their phosphorylated entities, respectively) in the model plant *A. thaliana*, by comparison with the LC–MS/MS method (Verstraete *et al*., 2020), which served as the reference method. These analyses revealed that the turbidimetric yeast assays enabled *in planta* identification of vitB1 accumulation and can pinpoint high thiamin plant lines as determined by the reference method. Moreover, combination of multiple assays allows an insight into the accumulation of biosynthetic intermediates. The metabolite levels determined via the assays often depict a discrepancy as compared to the LC–MS/MS reference method and should, therefore, be considered semi‐quantitative. This study demonstrates the potential that lies in the application of a carefully chosen panel of *S. cerevisiae* assays to estimate the levels of thiamin and its biosynthetic intermediates in plant samples, whether or not genetically engineered.

## Materials and Methods

### Plant material

Analyses (both LC–MS/MS and microbiological) were performed on *A. thaliana* (Colombia‐0) plant material, unless stated otherwise. Plants were grown under 16 h : 8 h, light : dark regime (150 µmol m^−2^s^−1^, white light) at 21°C on solidified half strength Murashige & Skoog medium (Murashige & Skoog, 1962) supplemented with 10 g l^−1^ sucrose.

In the case of plant material used for metabolite‐spiked samples, complete seedlings were harvested after 15 d of growth on thiamin‐free half strength Murashige & Skoog medium. For the analysis of thiamin‐supplemented plants, true leaf material was harvested after 35 d of *in vitro* growth on half strength Murashige & Skoog medium plates with or without 1 µM of thiamin. This concentration was previously shown to have an effect on the thiamin content of Arabidopsis plants (Pourcel *et al*., 2013). Analysis of metabolically engineered lines (Strobbe *et al*., 2021b) was performed on complete seedlings, harvested after 15 d of growth on thiamin‐free half strength Murashige & Skoog medium. Additionally two thiamin biosynthesis mutants, *tpk1*/*tpk2* (Ajjawi *et al*., 2007a) and *pale green1* (Hsieh *et al*., 2017), which lacks TH2 activity (Mimura *et al*., 2016; Hsieh *et al*., 2017), were included in this analysis. Arabidopsis *tpk1*/*tpk2* double mutants and *pale green1* mutant line were kindly provided by Prof. David Shintani and Prof. Ming‐Hsiun Hsieh, respectively. The engineered lines are indicated as ‘THIC’, ‘THI1’, ‘TT’ and ‘TTT’, referring to the single‐gene (THIC and THI1) and multi‐gene (TT (*THIC* and *THI1*) and TTT (*THIC*, *THI1* and *TH1*)) engineering approaches. The engineering strategies as well as their outcome and discussion are part of a previous metabolic engineering study (Strobbe *et al*., 2021b).

### LC–MS/MS analysis

For a detailed description, we refer to Verstraete *et al*. (2020), where we described the development and validation of an LC–MS/MS procedure to determine thiamin, its intermediates and phosphate derivatives in *A. thaliana*. A summary of the applied sample preparation procedure is given in Supporting Information Fig. S1. In short, the LC–MS/MS method allows accurate quantification of thiamin, TMP and TPP as well as HMP and HET. Furthermore, the phosphorylated equivalents of the pyrimidine (HMP‐PP) and thiazole (HET‐P) entities are assessed by comparing measurements in phosphatase treated and nontreated samples (split in two). Note that the methodology is not able to distinguish between HMP‐P and HMP‐PP; their sum is measured as phosphorylated HMP. The method was fully validated according to international guidelines (Verstraete *et al*., 2020), and has shown its applicability in characterization of engineered transgenic Arabidopsis lines (Strobbe *et al*., 2021b).

### Yeast assays

The yeast assays were performed according to an adapted protocol based on the described microbiological assay utilizing the *S. cerevisiae thi4* strain (Kall, 2003; Raschke *et al*., 2007; Mangel *et al*., 2017). The practical execution, considering both the plant extracts and the different yeast strains used, is comprehensively described in the Methods S1–S5. A schematic representation of the practical execution of the microbiological assays is shown in Fig. S2, while the experimental setup used to analyze metabolite‐spiked plant extract is shown in Fig. S3.

### Data analysis

Data were analyzed using Excel software v.16.0 (Microsoft Corporation, Redmond, WA, USA). A Shapiro–Wilk test was applied to determine the normality of the data, in order to decide whether a parametric (in case of normality) or nonparametric test should be applied on the data. To evaluate whether differences among multiple groups were significant, ANOVA (in the case of normal distribution of data) or a Kruskal–Wallis test (in the case of nonnormal distribution) was performed, followed by *post‐hoc* Tukey (ANOVA) or a Dunn's test (Kruskal–Wallis). Upon examination of the engineered lines, however, statistically significant differences as compared to the wild‐type, was assessed by using an unpaired, two‐sided *t*‐test (in case of normality) or a Mann–Whitney U test (nonparametric, in case of nonnormality). The *t*‐tests were preceded by an *F*‐test to determine homoscedasticity or heteroscedasticity. The engineered lines were analyzed using three biological replicates, which were then compared to wild‐type, which consisted of six biological replicates. Statistically significant differences in the figures were indicated by a single asterisk (*P* < 0.05) or double asterisks (*P* < 0.01). Indicated values depict mean ± SE. Violin plots were generated using free source Rstudio software v.1.4.1106 (RStudio^®^, Boston, MA, USA).

## Results

### Multiple thiamin auxotrophic mutants show a dose‐dependent response to thiamin supplementation

**Fig. 2:**
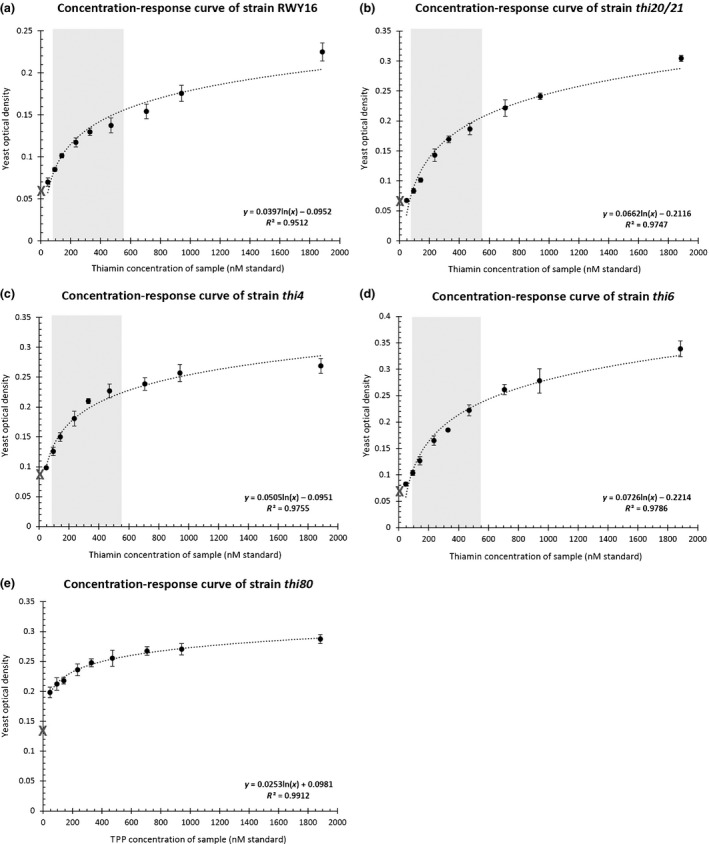
Dose–response curves for yeast microbiological thiamin assays. Dose–response curves are depicted for all five auxotrophic strains, being RWY16 (a), *thi20/21* (b), *thi4* (c), *thi6* (d) and *thi80* (e). The microbiological assays show a clear correlation (*R* ^2^ > 0.95) between the measured yeast optical density (OD) and the thiamin concentration added in the assay (standard). The OD values represent direct measurements from the TECAN Infinite 200 Pro plate reader (not corrected for path length) and should only be used for relative comparison. The gray crosses on the *y*‐axes indicate the OD values corresponding to 0 nM vitamin B1 (vitB1) standard (not included in the trendlines). Values represent the mean ± SE of four technical replicates. Dose–response curves (equations) are considered to enable estimation of metabolites from unknown samples, based on the relation between the OD and thiamin concentration, provided by the equation, expressed as molar thiamin equivalent (MTE) (e.g. for *thi6*: MTE = e^(OD+0.2214)/0.0726^). The MTE value corresponds to the amount of metabolites having the same growth restoring capacity as that amount of thiamin (expressed in nmol g^−1^). The range in which such a relation is optimal is indicated in gray.

Implementation of yeast in a microbiological assay panel to estimate the level of vitB1 and/or vitB1‐related metabolites relies on the availability of thiamin auxotrophic strains. These include *S. cerevisiae* strains harboring one or multiple mutations in crucial steps of the thiamin biosynthesis *de novo* (or salvage), therefore requiring thiamin (or biosynthetic intermediates) for their growth. Several thiamin auxotrophic yeast strains have been described, including RWY16 (*thi5/thi11/thi12/thi13* quadruple mutant, affected in four members of the *THI5* gene family, functionally redundant in terms of HMP formation (Wightman & Meacock, 2003)), *thi20/21* (affected in the phosphorylation of HMP‐P) (Llorente *et al*., 1999; Kawasaki *et al*., 2005), *thi4* (affected in the synthesis of the thiazole moiety) (Raschke *et al*., 2007; Chatterjee *et al*., 2011), *thi6* (affected in the condensation of pyrimidine and thiazole moieties to form vitB1) (Nosaka *et al*., 1994) and *thi80* (affected in the synthesis of TPP) (Nosaka *et al*., 1993). In the latter, thiamin pyrophosphokinase activity (THI80), was described to be lowered, but not abolished (Nishimura *et al*., 1991). These mutant *S. cerevisiae* strains were acquired (Table S1) and their growth was examined in response to thiamin or TPP (in the case of the *thi80* strain), by measurement of the optical density (OD). The five strains examined all depicted a clear correlation (*R* ^2^ > 0.95) between growth and the concentration of thiamin (or TPP in the case of strain *thi80*) in the medium (Fig. 2). However, the variation in OD observed with the *thi80* (Fig. 2e) strain was much lower as compared to the other strains, since it could grow better in the absence of vitB1. The *thi80* strain was, therefore, not further evaluated in this work. Strains RWY16, *thi20/21*, *thi4* and *thi6*, however, proved to be well‐suited candidates to serve in a microbiological assay panel, given their distinct responsiveness to varying levels of thiamin (Fig. 2a–d). In general, these assays should ideally be applied in the linear to early logarithmic phase of the growth response curve, as this would ensure the highest sensitivity and still allow assessment of up to five‐fold changes in vitB1 levels (gray zones in Fig. 2a–d). The observations confirm the ability of the different strains to respond to thiamin in a dose‐dependent manner (Nishimura *et al*., 1991; Nosaka *et al*., 1994; Wightman & Meacock, 2003; Kawasaki *et al*., 2005; Raschke *et al*., 2007). Assessing these strains in parallel corroborate the potential usage of these strains in microbiological assays, as a promising extension of the described assay utilizing *thi4* (Raschke *et al*., 2007).

### Yeast assays to identify increased metabolite levels in spiked plant extracts

**Fig. 3:**
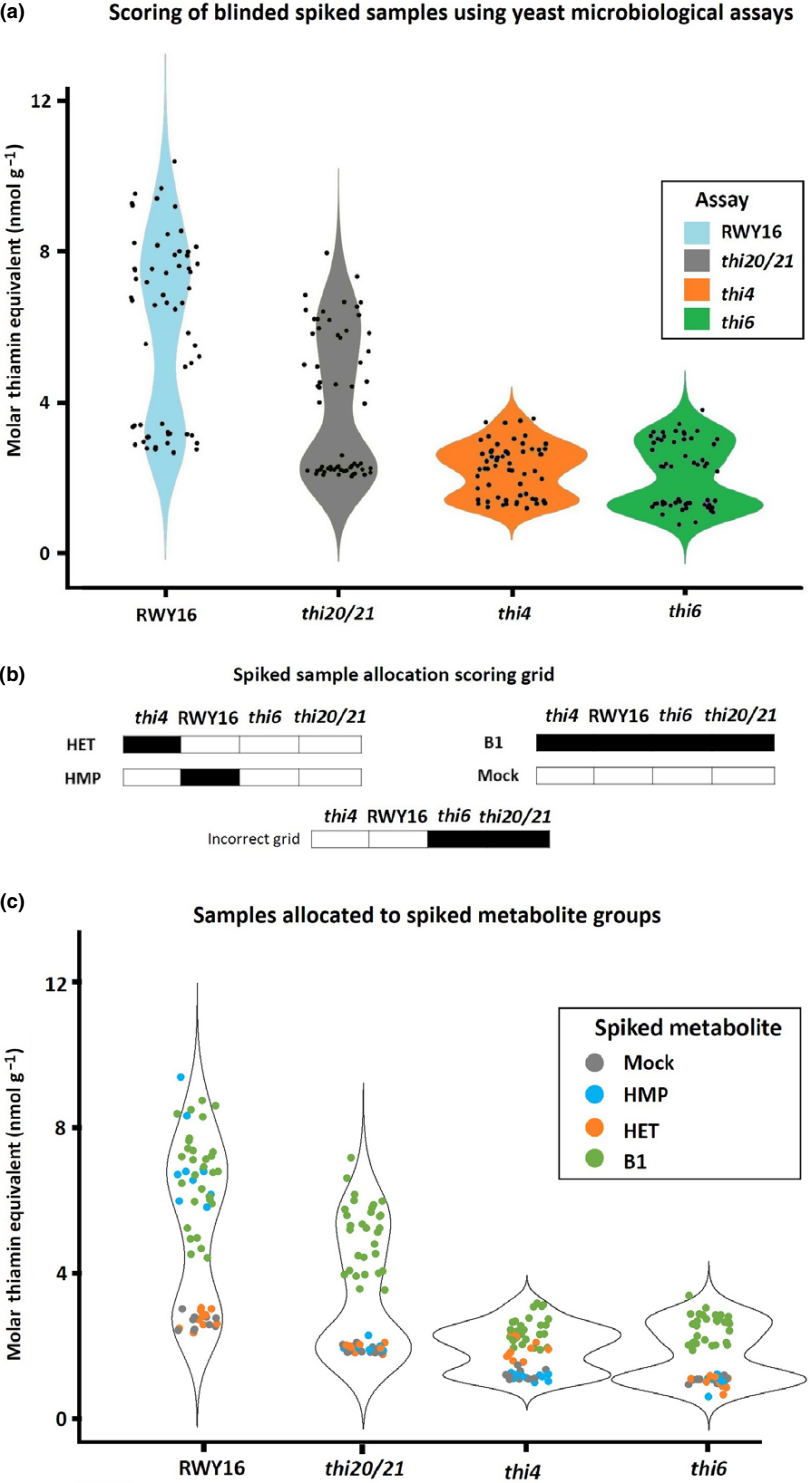
Assessment of the ability of the yeast assays to detect thiamin‐related metabolites by the analysis of spiked plant extracts. Arabidopsis plant extracts were spiked with either HMP, HET, TMP, thiamin, TPP or nonspiked (mock). The resulting 60 samples, 10 samples per spiking condition, were independently coded to allow blind analysis. (a) By applying the four different yeast assays (RWY16, *thi20/21*, *thi4* and *thi6*), the blind‐coded samples could be divided into a low and high metabolite group for each assay, as shown in the violin plots (raw data in Supporting Information Fig. S5). Violin plots were generated using free source Rstudio software v.1.4.1106 making use of the ggplot2 data visualization package. The width of the ‘violin’ plot reflects the distribution of the data, indicating that two groups can be distinguished. (b) Representation of the ‘scoring grid’, established based on the genetic background and hence the list of rescuing metabolites of the autotrophic strains (Fig. 1b), which may allow to predict with which specific metabolite a sample was spiked. White grid sections represent absence of restored growth, while black represents prototrophic growth of the specific yeast strain. Based on the hypothesized list of growth restoring metabolites (Fig. 1b), certain patterns of growth restoration/nonrestoration are possible, whilst others are expected to not occur (e.g. if *thi6* or *thi20/21* are restored, RWY16 and *thi4* should also be restored, hence the incorrect grid). Allocation of the samples to certain groups can be achieved by examining the sample by all four assays and allocating them within a high metabolite or low metabolite group, represented by black and white in the scoring grid respectively. The results of the separate yeast assays on all 60 samples are presented in Fig. S5, while the application of the scoring grid on the complete list of samples is shown in Supporting Information Table S2. (c) Application of the scoring grid on the complete sample set (Table S2) allowed to correctly identify the spiked sample subgroups (mock, HMP, HET and vitB1). Abbreviations: HMP, 4‐amino‐2‐methyl‐5‐hydroxymethylpyrimidine; HET, 4‐methyl‐5‐β‐hydroxyethylthiazole; TMP, thiamin monophosphate; TPP, thiamin pyrophosphate.

**Fig. 4:**
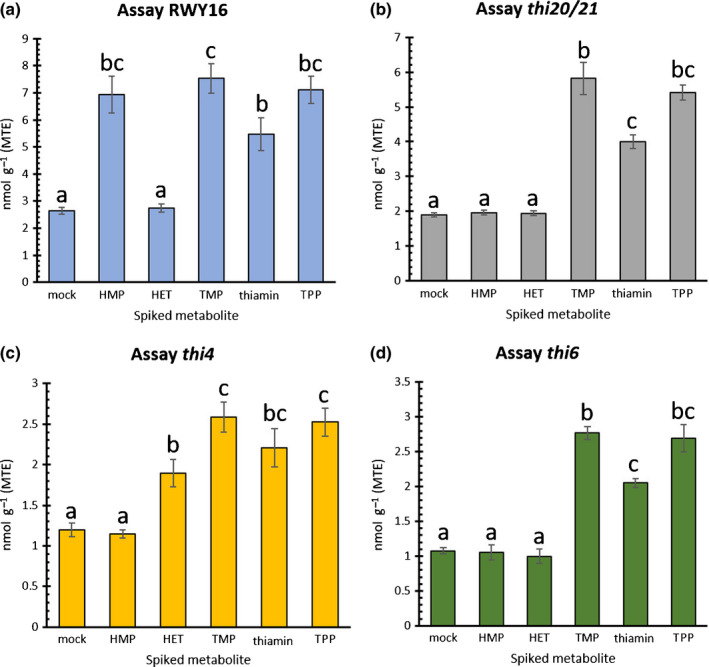
Effect of spiking a particular metabolite on the results of the different yeast assays. The measured MTE (molar thiamin equivalent) values upon spiking with the different thiamin‐related metabolites are depicted for yeast assay RWY16 ((a) can be rescued by HMP‐PP, TMP, thiamin and TPP), *thi20/21* ((b) can be rescued by HMP‐PP, TMP, thiamin and TPP), *thi4* ((c) can be rescued by HET‐P, TMP, thiamin and TPP) and *thi6* ((d) can be rescued by TMP, thiamin and TPP). Bars indicate the mean ± SE of 10 samples, whether or not spiked with a particular metabolite. Significant differences were determined via nonparametric tests, as the experiments were found, using Shapiro–Wilk test, to have one or multiple nonnormal distributed datasets. Kruskal–Wallis test revealed presence of significant differences within the groups, which were identified using *post hoc* Dunn's test. Different lowercase letters indicate significant differences between groups (*P* < 0.05). Abbreviations: HMP‐PP, 4‐amino‐2‐methyl‐5‐hydroxymethylpyrimidine ((pyro)phosphate); HET‐P, 4‐methyl‐5‐β‐hydroxyethylthiazole (phosphate); TMP, thiamin monophosphate; TPP, thiamin pyrophosphate.

Given the different genetic backgrounds of the yeast strains, each mutant line is predicted to be rescued by a distinct set of metabolites, downstream of the defective enzymatic function (see biosynthesis scheme in Fig. 1a, and hypothesized growth restoring metabolites in Fig. 1b). For example, strain *thi6* is the only strain which is hypothesized to specifically respond to vitB1 (thiamin, TMP and TPP), while the other strains are presumably also rescued by at least one biosynthetic intermediate (Fig. 1b). Therefore, an experiment was set up aimed at examining the collective ability of the different yeast assays employing the set of available mutants to identify plant samples with enhanced levels of different metabolites. The experiment consisted of making a bulk sample of plant material followed by preliminary assessment of vitB1 content using the *thi6* yeast assay (Fig. S3; Methods S6). The resulting estimation of vitB1 content, calculated based on the relation between OD and thiamin concentration (‘molar thiamin equivalent (MTE)’) (Fig. 2d) (*thi6* microbiological assay), was set as baseline vitB1 level of the bulk plant material. Subsequently, this bulk material was divided into 60 separate samples, to which thiamin‐related metabolites; HMP, HET, thiamin, TMP or TPP, were added. All metabolites were spiked at the same concentration to the plant extracts, in such a way that the MTE baseline level was surmounted by three‐fold. This spiking level was based on differences obtained in genetically engineered samples (see further; Strobbe *et al*., 2021b) and was considered to be low enough to impose a challenge when aiming at distinguishing spiked from nonspiked samples. This yielded six sample sets (mock (i.e. nonspiked), HMP, HET, thiamin, TMP and TPP), each consisting of 10 samples, which were randomized prior to blind‐coded analysis. The application of each assay on the complete sample set resulted in the allocation of the samples into two groups for that specific assay; a group with low (baseline) and a group with higher MTE (Figs 3a, S4). Through the application of a ‘scoring grid’ (Fig. 3b), which was established based on the proposed list of rescuing metabolites (Fig. 1b; based on knowledge of the genetic background of the auxotrophic strains), the specific metabolite with which a certain sample was spiked could be predicted. For example, HET spiked samples are expected to result in higher MTE for *thi4* assay, while depicting baseline MTE in RWY16, *thi20/21* and *thi6* assays. Samples spiked with vitB1 (thiamin, TMP or TPP), however, are expected to display increased MTE in all four microbiological assays (Fig. 3b). Note that HMP‐PP spiked samples would be expected to result in a higher MTE for the *thi20/21* assay (as well as the RWY16 assay), however, HMP‐PP could not be included in the spiking experiment as this compound is not commercially available. The screening of the complete sample set using the established scoring grid (shown in Table S2) allowed us to correctly allocate all 60 (blind‐coded) samples into four groups (mock, HMP, HET and vitB1 spiked) (Fig. 3c). Although no distinction could be made between the different B1 vitamers spiked, a clear insight into the presence of the specific pyrimidine (HMP) and thiazole (HET) vitB1 biosynthetic intermediates could be obtained. After samples were correctly allocated to the different groups, the effect of each metabolite on the result of the specific yeast assay could be examined. Spiking samples with nonrescuing metabolites (e.g. HMP or HET to *thi6*) resulted in MTEs which were indistinguishable from baseline in all assays tested (Figs 4, S4), while rescuing metabolites, downstream of the lost enzymatic step, did result in a significant increase in the retrieved MTE (Figs 4, S4). The different assays did report a difference in absolute MTE measured, as shown by the different scale of the *Y*‐axis in the different panels of Fig. 4. Moreover, it should be noted that HET (and to a lesser extent, thiamin) had a relatively lower impact on the observed MTE as compared to TMP and TPP (and HMP in RWY16). Samples spiked with fresh stock solutions of the metabolite set were subsequently tested (Fig. S4), to confirm that this effect is not caused by variation in stock dilutions. The discrepancy in MTE values obtained from the same molar concentrations of metabolites indicates that these metabolites exhibit a different power to restore growth of the yeast cells. In this way, the *thi4* assay seems to have a slightly lower sensitivity for HET. The results obtained from this experiment underscore that application of the yeast assay panel, when executed and interpreted in a conjoint manner, allows insight into whether different levels of vitB1, as well as its biosynthetic intermediates, are present in spiked plant material.

### Yeast assays are able to identify *in vivo* thiamin accumulation in *A. thaliana*

**Fig. 5:**
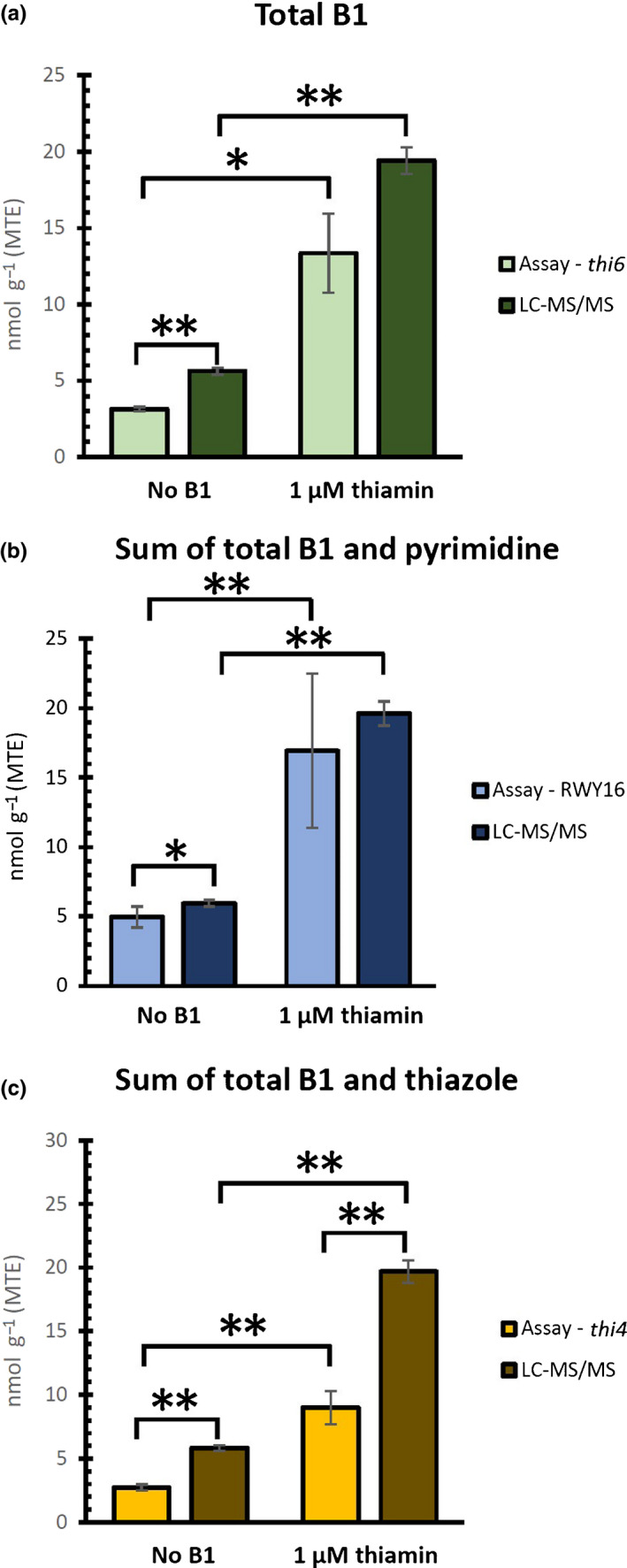
Different yeast assays allow observing an increase in thiamin‐related metabolites *in planta* upon thiamin supplementation. Leaf material of Arabidopsis plants, grown on half strength Murashige & Skoog medium with or without 1 µM of thiamin for 35 d, was used to test the ability of the yeast assays to distinguish *in planta* differences in thiamin‐related metabolites. Based on the knowledge of *Saccharomyces cerevisiae* thiamin biosynthesis genes presented in Fig. 1, strain *thi6* is considered to allow estimation of the total vitamin B1 (vitB1) content of plant samples. Similarly, utilizing strains RWY16 and *thi4* in yeast assays may provide information regarding the sum of total vitB1 and pyrimidine and the sum of total vitB1 and thiazole, respectively. The metabolite levels acquired via the liquid chromatography–tandem mass spectrometry (LC–MS/MS) reference method are expressed in nmol g^−1^, while the metabolite levels of the yeast assays are also expressed as nmol g^−1^ (MTE, molar thiamin equivalent), thereby allowing adequate comparison. (a) Levels of total vitB1 as estimated by the *thi6* assay, as well as quantified by LC–MS/MS. (b) Yeast assay RWY16 was used to estimate the sum of total vitB1 and pyrimidine, and compared to LC–MS/MS results. (c) Yeast assay *thi4* was used to estimate the sum of total vitB1 and thiazole, and compared to LC–MS/MS results. Mean values ± SE of five biological replicates are shown. The datasets were found to depict normal distribution, as verified via Shapiro–Wilk test. Statistically significant differences were detected via two‐sided *t*‐test (scedasticity depending on the outcome of preceding *F*‐test) and are indicated by a single asterisk (*P* < 0.05) or double asterisks (*P* < 0.01).

The analysis of spiked plant extracts showed that the utilization of different yeast assays allowed the relative estimation of thiamin and thiamin‐related metabolites in tested samples. However, the question remains whether or not these assays can detect alterations in actual metabolite levels *in planta*. Therefore, Arabidopsis plants were grown on medium containing thiamin (1 µM), known to alter the plant thiamin content *in vivo* (Pourcel *et al*., 2013). The resulting plant material was analyzed via the aforementioned yeast assays as well as with the validated LC–MS/MS method (Verstraete *et al*., 2020), which served as the reference method. For both methodologies, the metabolite content is expressed as nmol g^−1^ of plant material, which is further specified as MTE for the yeast assays, since all were calibrated based on a thiamin standard (Fig. 2). While both the yeast assays and the LC–MS/MS analysis confirmed the increased vitB1 content *in vivo* (Fig. 5), the resulting quantitative data differed between both methodologies. This became evident when comparing the LC–MS/MS data with those obtained by the different yeast assays (total vitB1 for the *thi6* assay; sum of vitB1 and pyrimidine for the RWY16 yeast assay, sum of vitB1 and thiazole for the *thi4* assay) (Fig. 5). However, a relative comparison of supplemented (1 µM thiamin) and nonsupplemented plants resulted in the same conclusion when analyzed using the assays or the LC–MS/MS reference method, namely a three‐ to five‐fold increase in metabolite levels upon growth on thiamin. Here, metabolite level refers to the specific sum of the set of metabolites measured, depending on the microbiological assay used, as compared to their LC–MS/MS counterpart. These results show that, though not providing similar absolute quantification (exact nmol g^−1^ amount), the relative comparison of metabolite levels using the microbiological assays allowed equal conclusions as the LC–MS/MS reference method, enabling adequate detection of *in planta* metabolite increases. Note that the microbiological assay using *thi20/21* (Fig. S6) has no specific LC–MS/MS reciprocal, as the LC–MS/MS methodology is not capable of distinguishing between the different phosphorylated pyrimidine entities (HMP‐P and HMP‐PP) (Verstraete *et al*., 2020). In conclusion, while the yeast microbiological assays are capable of detecting *in planta* vitB1 alterations, they provide relative rather than absolute metabolite information.

### Yeast microbiological assays can provide a crude profiling of vitB1 and its biosynthetic intermediates in transgenic Arabidopsis lines

**Fig. 6:**
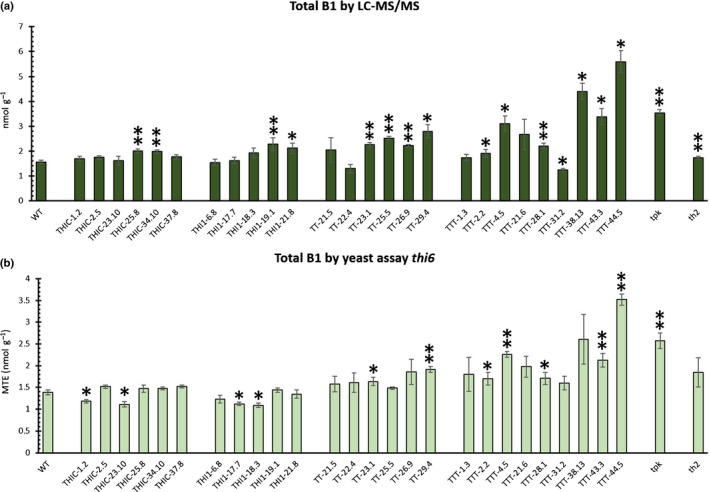
Comparison of total vitamin B1 (vitB1) levels in genetically engineered Arabidopsis lines, measured by the *thi*6 yeast assay and liquid chromatography–tandem mass spectrometry (LC–MS/MS) method. The engineered lines overexpressing *AtTHIC* (THIC), *AtTHI1* (THI1), both *AtTHIC* and *AtTHI1* (TT) or *AtTHIC*, *AtTHI1* and *AtTH1* (TTT), described by Strobbe *et al*. (2021b), were utilized. Mean values ± SE of three (transgenic) or six (WT) biological replicates are shown. (a) Levels of total vitB1 (nmol g^−1^; sum of thiamin, TMP and TPP) measured via LC–MS/MS after phosphatase treatment. (b) Levels of total vitB1 (MTE), measured by *thi6* yeast assay. The datasets were tested for normality via Shapiro–Wilk test. In case of normality, statistically significant differences were detected via two‐sided *t*‐test (scedasticity depending on the outcome of preceding *F*‐test). In case of nonnormality, Mann–Whitney U test was used. Significant differences, by comparison to WT plants, are indicated by a single asterisk (*P* < 0.05) or double asterisks (*P* < 0.01). Abbreviations: WT, wild‐type; TMP, thiamin monophosphate; TPP, thiamin pyrophosphate; MTE, molar thiamin equivalent.

The ability of the different yeast assays to detect *in vivo* variations of vitB1 content as well as their responsiveness towards different sets of metabolites (sum of vitB1 and pyrimidine for RWY16; sum of vitB1 and thiazole for *thi4*), raises the question whether these assays can be applied to estimate the content of vitB1 and its biosynthetic intermediates in different plant genetic backgrounds. To explore this, an array of genetically altered Arabidopsis lines was employed. Lines originating from an earlier metabolic engineering strategy (Strobbe *et al*., 2021b), which aimed at elevating the vitB1 content in Arabidopsis plants, were used. This array further included thiamin biosynthesis mutants *tpk* (*tpk1/tpk2* double mutant (Ajjawi *et al*., 2007a)) and *th2* (Mimura *et al*., 2016; Hsieh *et al*., 2017). The *tpk* and *th2* lines are an interesting addition to the array of engineered lines as they have been described to have altered thiamin metabolism and both can be maintained *in vitro* until sampling at 15 d of age, despite their aberrant growth on soil in the absence of TPP supplementation. The results endorse that lines with aberrant B1 content can be detected with the yeast assays, as verified with the LC–MS/MS method. LC–MS/MS analysis confirmed the altered vitB1 status of the analyzed lines, reaching an approximately three‐fold increase over wild‐type in high vitB1 lines (Fig. 6a). Assessing the vitB1 status by utilization of the *thi6* yeast microbiological assay (Fig. 6b) confirmed the pattern observed from the LC–MS/MS analysis (Fig. 6a), although absolute values differed. Indeed, the yeast *thi6* assay was able to accurately identify high vitB1 lines harboring an above 1.5‐fold increase in vitB1 content (Fig. S7). This was also consistent for the *thi20/21* yeast assay (Fig. S6b), indicating that in these samples *thi20/21* also estimated total vitB1 levels. This, however, does not give any indication that the *thi20/21* assay is unable to detect HMP‐PP (Fig. S8a), as HMP‐PP is not commercially available and the LC–MS/MS method is unable to distinguish the different phosphorylated HMP entities.

**Fig. 7:**
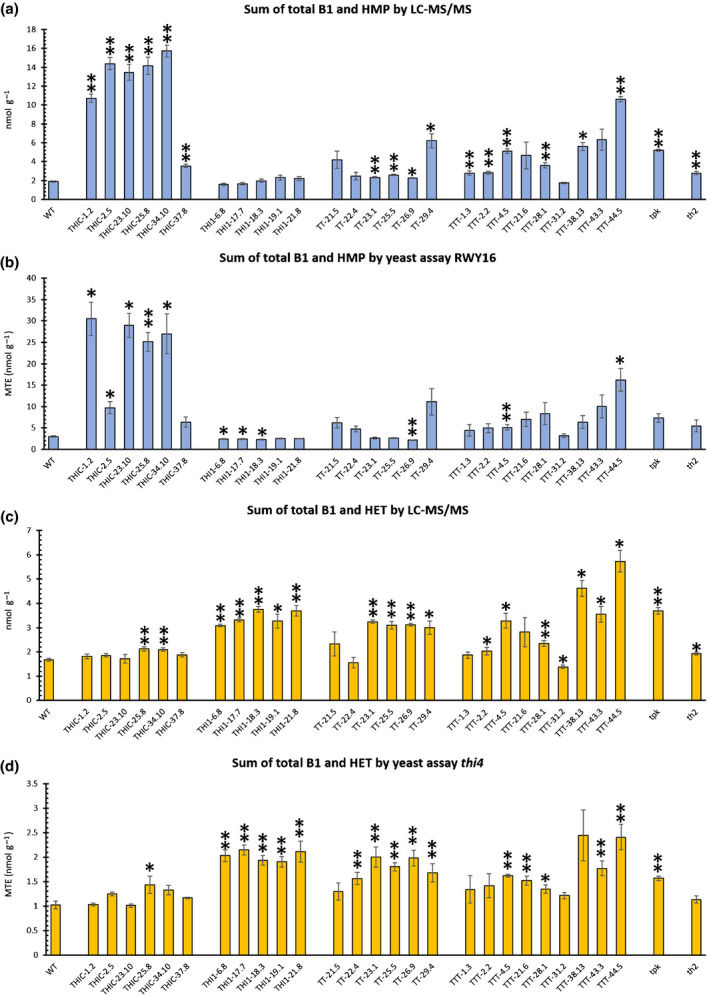
Comparison of the sum of total vitamin B1 (vitB1) and its intermediates in genetically engineered Arabidopsis lines, measured by the RWY16 or *thi4* yeast assay vs liquid chromatography–tandem mass spectrometry (LC–MS/MS) method. The engineered lines overexpressing *AtTHIC* (THIC), *AtTHI1* (THI1), both *AtTHIC* and *AtTHI1* (TT) or *AtTHIC*, *AtTHI1* and *AtTH1* (TTT), described by Strobbe *et al*. (2021b), were utilized. Mean values ± SE of three (transgenic) or six (WT) biological replicates are shown. (a) Sum of total pyrimidine and total vitB1 levels (nmol g^−1^) as measured by LC–MS/MS. Levels of total vitB1 and total pyrimidine were obtained by quantifying thiamin and HMP levels, respectively, after phosphatase treatment. (b) Total vitB1 and pyrimidine levels (MTE), as measured by the RWY16 yeast assay. (c) Sum of total thiazole and total vitB1 (nmol g^−1^) as measured by LC–MS/MS. Levels of total vitB1 and total thiazole are obtained by quantifying thiamin and HET levels, respectively, after phosphatase treatment. (d) Total vitB1 and thiazole levels (MTE) as measured by the *thi4* yeast assay are shown. The datasets were tested for normality via Shapiro–Wilk test. In case of normality, statistically significant differences were detected via two‐sided *t*‐test (scedasticity depending on the outcome of preceding *F*‐test). In case of nonnormality, Mann–Whitney U test was used. Significant differences, by comparison to WT plants, are indicated by a single asterisk (*P* < 0.05) or double asterisks (*P* < 0.01). Abbreviations: WT, wild‐type; HMP, 4‐amino‐2‐methyl‐5‐hydroxymethylpyrimidine; HET, 4‐methyl‐5‐β‐hydroxyethylthiazole; MTE, molar thiamin equivalent.

Moreover, assessment of the engineered lines using RWY16 and *thi4* assays demonstrated that these assays predict the accumulation of combined vitB1 and pyrimidine or thiazole, respectively (Fig. 7). Interestingly, the combination of these assays could provide identification of lines exhibiting high accumulation of the biosynthetic intermediates. Theoretically, a rough estimation of both the pyrimidine and thiazole intermediates can be obtained by subtracting the RWY16 and *thi4* yeast assay MTE values, respectively, with the MTE results measured in the *thi6* assay (Fig. 1b). Indeed, the latter approach allowed us to pick up the high accumulation of pyrimidine (Fig. S9) and/or thiazole (Fig. S10) in several engineered Arabidopsis lines. Finally, these results show that deploying a carefully designed panel of yeast assays can be used to perform vitB1 profiling in an array of different genetic backgrounds and even holds the potential to indicate high intermediate accumulating lines.

## Discussion

Within this study, we comprehensively evaluated the potential of applying yeast assays to assess the content of vitB1 and its intermediates in plants, using *A. thaliana* as a reference. The results, as compared to LC–MS/MS data, revealed the suitability of the proposed assay panel to serve as a simple and inexpensive alternative to allow the rapid screening of thiamin metabolite levels in plant material.

### Evaluation of the extended yeast assay procedure

This study involves assessment of the value of including additional biosynthesis mutants of *S. cerevisiae* to extend the previously used *thi4* microbiological assay (Raschke *et al*., 2007; Mangel *et al*., 2017), to more accurately measure (a specific set of) metabolites of interest, whilst comparing their potential to the validated LC–MS/MS reference method (Verstraete *et al*., 2020). The *thi80* mutant did not display a severe TPP dependent growth, making it an unsuitable candidate to be used in microbiological assays aimed at quantifying this B1 vitamer. This further hints that the *thi80* strain is indeed not a null mutant, and still exhibits at least some thiamin pyrophosphokinase activity. Usage of the other different yeast mutants, each deprived of a specific enzymatic activity in vitB1 biosynthesis (RWY16, *thi20/21*, *thi4* and *thi6*), allowed us to distinguish between total vitB1, pyrimidine and thiazole moieties. As expected, the growth of the different strains could only be enhanced by all products downstream of the mutated enzymes (Figs 1b, 4). By doing so, the previously undesired sensitivity towards the vitB1 biosynthetic intermediates can be turned into an advantage by carefully selecting different mutant strains and comparing their results in microbiological assays. Although with this approach we could obtain an estimation of total vitB1 (Fig. 6b), HMP (by comparison of *thi6* and RWY16) and HET (by comparison of *thi6* and *thi4*) levels in transgenic plant samples (Figs 5, 6, respectively), the comparison of data with those obtained from LC–MS/MS analysis indicates that the yeast assays provide semi‐quantitative results, serving comparative purposes. Since thiamin was used to calibrate all assays, all measurements refer to MTE, and biological activities may differ across the different analytes, the data cannot be considered as absolute and should rather be seen as valuable estimates. From Fig. 4(c), it could be derived that HET, while able to restore and enhance the growth of *thi4*, harbored a lower activity compared to thiamin, although spiked at the same molar concentration. Therefore, based on these differences in activities, HET levels could be considered slightly underestimated. Nevertheless, similar trends in metabolite changes upon engineering could be discerned in the transgenic lines, irrespective of the applied assay, demonstrating the feasibility of the yeast assay panel to support the screening of a variety of lines to assess variation of vitB1 and its biosynthetic intermediates. This demonstrates that, despite serving semi‐quantitative analysis only, the assays can pinpoint high‐metabolite lines and provide an idea about the fold enhancement reached. The combined use of the yeast assays provides a simple tool to pinpoint changes (increase/decrease) in thiamin metabolite levels, which require interpretation in a semi‐quantitative manner. In summary, these results illustrate that screening data using microbiological assays, while valuable, should always be confirmed using highly accurate methods, such as LC–MS/MS, to obtain a quantitatively reliable profile of the thiamin (or related metabolites) content in plants.

### Comparison of the microbiological assay vs LC–MS/MS

Every analytical approach is accompanied by advantages and disadvantages, as illustrated in Table 1 and already briefly touched upon earlier, and they should all be evaluated on a case‐by‐case basis, taking into account the warranted application of the analytical technique. While the lack of required specialized equipment using yeast assays can definitely be considered an advantage in terms of cost of the analysis to screen plant samples for their thiamin profile, it may come at the expense of sample throughput. However, the implementation of a multiple‐well plate reader, as applied in this study, also ensures that high‐throughput turbidimetry‐based screening is feasible. Although this adds to the cost of the assays, the required investment for a plate reader is still approximately 10‐fold lower compared to the purchase of an LC–MS/MS instrument, as the latter can easily cost around 200 000 euros. The microbiological assays also demand sterile laboratory conditions, requiring an autoclave as well as a sterile flow cabinet, both of which can cost up to a few thousand euros. This gives an indication that, considering required equipment, microbiological assays are an order of magnitude cheaper as compare to their LC–MS/MS counterpart.

**Table 1:** Comparison of microbiological and liquid chromatography–tandem mass spectrometry (LC–MS/MS) analysis to evaluate the profile of thiamin and its intermediates in food products.

	Microbiological assay	LC–MS/MS
Cost	+	+++
Specificity	+	+++
Sensitivity	+	+++
Time commitment	++	++
Accuracy	+	+++
Measurement of activity	+	−
Required experience	+	+++
Interferences	+	−
Standardization	−	+
Sample throughput	+++	+
Sterile conditions	+	−
Multi‐assay procedure	+	−

Here, both the microbiological assays as well as the LC–MS/MS method are evaluated based in different criteria, in which a minus sign represents the lowest score, while (multiple) plus signs indicate ascending higher scores. Note that higher scores are not always beneficial, as for cost, time commitment, required experience, interferences and sterile conditions a lower score would be preferred.

Both methodologies, microbiological and LC–MS/MS, involve a time‐consuming incubation step, i.e. 17 h incubation of the yeasts with the plant extracts and 24 h incubation of the samples to allow phosphatase treatment, respectively. However, the actual hands‐on‐time of both methods is relatively limited. The chromatographic separation of the thiamin‐related metabolites from other constituents in the plant extracts limits the risk of interferences, thereby improving the accuracy of the results. While total vitB1, HMP and HET levels can be quantified in one analytical run using LC–MS/MS analysis, multiple microbiological assays using different yeast strains (RWY16, *thi6* and *thi4*) should be combined to estimate the levels of vitB1 intermediates. Given that the yeast assays are biologically based, this methodology has the advantage of being able to detect a whole set of biologically active compounds, independent of their chemical structure. However, this advantage comes at the price of a lower specificity and accuracy. A good illustration thereof involves the inability of the yeast assays to distinguish between different B1 vitamers (TMP, thiamin, TPP), which is feasible using the LC–MS/MS method. Another potential downside of the turbidimetric assays involves their reliance on sufficient stability of the metabolite during the relative long (17 h) growth of the yeast cells. Furthermore, it is important to indicate that the yeast assays exhibit a limited range within which the metabolites can be assessed, corresponding to the linear range of the dose–response curves (Fig. 2). This was seen in diluted samples, in which clear drops in MTE values were more clearly observed when the original samples depicted high MTE values, indicating dilutions are only appropriate when the samples were at the higher end of the linear range (Fig. S8b, the *tpk* sample being an exception). This highlights the need to assure (via dilution) that the measured OD values are within the acceptable concentration range as indicated on the growth curves (Fig. 2).

Although the results generated by the yeast assays were reproducible, LC–MS/MS analysis has the possibility of including a stable isotope‐labeled analog of the analytes of interest in the method, which may serve as an internal standard to compensate for any variation during the analytical procedure, and will consequently benefit the comparison of results across laboratories. The latter is important in the context of nutrition labeling of food products, whether or not fortified, where labels should represent the true nutritional value of a product when entering the market, irrespective of the analyzing laboratory. However, in an initial stage of food crop development, either through breeding or genetic engineering, the comparative analysis employing microbiological assays is fit‐for‐purpose as it may serve as a rapid screening tool. Since biofortification strategies mostly target populations in low‐ and middle‐income countries, the inexpensiveness and simplicity of microbiological assays, omitting the need for highly trained analysts, may stimulate local biofortification endeavors. This is of high importance, since biofortification interventions aim at providing self‐reliance to local populations.

### Application potential

Given the equipment and expertise required for precise vitB1 determination, only a limited number of groups can do so. Strikingly, *in planta* quantification of the thiamin biosynthetic intermediates has only been achieved very recently (Verstraete *et al*., 2020, 2021). The described turbidimetric yeast assays can, depending on the purpose, be a valid alternative to obtain relative vitB1 levels. The described yeast assays promise to be well suited for implementation in thiamin biofortification/metabolic engineering endeavors. The previously reported application of the *thi4* assay in screening of cassava lines (Mangel *et al*., 2017) as well as its use in scoring genetically engineered rice lines (Mangel, 2016) together with our results of both brown and polished rice seeds (Fig. S11), demonstrates the potential implementation of the assay in analysis of different plant tissues and/or species. In this regard, a fast, easy and cheap method for vitamin quantification facilitates the unraveling of genetic factors underlying thiamin accumulation. Examples include genome‐wide association studies (GWAS) of massively consumed crops such as wheat (*Triticum aestivum*) (Li *et al*., 2018), as well as orphan crops such as foxtail millet (*Setaria italica*) (Trivedi *et al*., 2018). Moreover, availability of trustworthy microbiological assays makes identification of elite, thiamin rich germplasm, such as thiamin rich potato lines (Goyer & Sweek, 2011), more easily achievable. These assays make measurement of vitB1 content more easily obtainable, which turns it more appealing as an additional parameter when assessing nutritional value of plant products.

On top of quantification of vitB1, being able to make a (rough) estimation of the biosynthetic intermediates, via the combined application of these assays, can be a valuable asset. The latter is particularly true in the case of metabolic engineering. Indeed, metabolic engineering approaches aim at overcoming metabolic hurdles, by over‐activation of rate‐limiting steps, and are often obstructed by insufficient downstream processing, consequently leading to accumulation of undesired intermediate products. Therefore, a quick scan of engineered lines to assess adequate metabolic flux towards the desired products, whilst limiting the build‐up of unwanted intermediates can be advantageous. Examples of such studies include the enhancement of vitB1 in Arabidopsis (Bocobza *et al*., 2013; Dong *et al*., 2015; Strobbe *et al*., 2021b), rice (Dong *et al*., 2016; Strobbe *et al*., 2021a) and *Lotus japonicus* (Yin *et al*., 2019). In some cases, high levels of these metabolites are to be avoided since not all compounds are generally regarded as safe (GRAS (generally recognized as safe) compounds). By doing so, the metabolic flux can be directed towards the desired metabolite, vitB1 in this case, thus limiting the metabolic burden of intermediate over‐production.

Although the combined application of the different microbiological assays on the transgenic lines has allowed us to estimate changes in levels of the pyrimidine and thiazole moieties (Figs S8, S9), these changes upon genetic engineering, compared to wild‐type, are relatively high. By contrast, when considering biofortification through breeding, changes may be more subtle due to the limited natural variation in thiamin and thiamin‐related metabolites. For these purposes, yeast microbiological assays will likely only be useful for the assessment of total vitB1, as the intermediates can be overshadowed since these metabolites typically only reach a fraction of the molar total vitB1 concentration (Strobbe *et al*., 2021a,b). Breeding of crops towards higher vitamin content has shown to be a successful method to ensure higher vitamin consumption by certain populations (Bouis & Saltzman, 2017). This methodology is, however, reliant on availability of appropriate germplasm and screening for desired variation in vitamin content (Van Der Straeten *et al*., 2020). As this could involve testing vast amounts of plant material, microbiological assays could provide a high‐throughput, inexpensive and crude method for screening of potential candidates as has been shown by Mangel and colleagues for cassava (Mangel *et al*., 2017). *Post hoc* verification, using trustworthy methodologies such as LC–MS/MS (Verstraete *et al*., 2020, 2021) at a later stage in the breeding process will remain necessary to confirm and acquire deeper insight into the metabolite profile, particularly relating to quantitative aspects.

Furthermore, on top of their applicability in screening for vitB1 content in different crop cultivars (Mangel *et al*., 2017) or following metabolic engineering strategies, these microbiological assays could prove useful in fundamental research (Raschke *et al*., 2007). In that perspective, yeast assays could be used to assess whether a specific stress response is accompanied by alterations in vitB1 levels, given that vitB1 levels are known to be influenced by both biotic and abiotic stress conditions (Wang *et al*., 2016; Kamarudin *et al*., 2017; Kartal & Palabiyik, 2019; Fitzpatrick & Chapman, 2020). Moreover, their ability to provide an indication of the accumulation of thiamin biosynthesis intermediates could make these assays useful in the characterization of thiamin biosynthesis genes from different species.

Finally, the described panel of different yeast strains can also be applied in the broader context of food analysis. Traditionally, microbiological assays have been applied to a variety of food products, including milk and other dairy products (Niven & Smiley, 1943). Although the estimation of the pyrimidine and thiazole content of foods does not reflect on their nutritional quality, it may provide insights into the stability of the B1 vitamers upon food processing.

In conclusion, our results show that the proposed panel of yeast assays can be used as an inexpensive, high‐throughput screening method of vitB1 content in plant tissues, and can even provide information on accumulation of thiamin intermediates. Future breeding and metabolic engineering strategies can utilize these methods in a pre‐screening phase, after which promising candidates can be verified using validated mass spectrometric analysis.

## Author contributions

DVDS, SS, JV and CS designed the experiments. TBF optimized the *thi4* assay protocol. MF, TFL and MMO generated mutant yeast strain *thi20/21*. SS collected plant material and conducted the microbiological assays. Molecular data analysis was done by SS, JV, CS and DVDS. JV operated LC–MS/MS and analyzed the LC–MS/MS data together with CS. SS, JV, CS and DVDS wrote the manuscript, all authors commented and approved the manuscript. DVDS conceived and coordinated the project.

## Supporting information


**Fig. S1** Schematic overview of the sample preparation procedure for the liquid chromatography–tandem mass spectrometry (LC–MS/MS) method.
**Fig. S2** Schematic representation of yeast microbiological assays for thiamin determination.
**Fig. S3** Schematic overview of blinded yeast assays using metabolite‐spiked plant extracts.
**Fig. S4** Repeat of spiking experiment.
**Fig. S5** Analysis of 60 different metabolite‐spiked plant extracts with different yeast assays.
**Fig. S6** Results of thi20/21 yeast assay applied on both Arabidopsis grown on thiamin and genetically engineered lines.
**Fig. S7** Assessing the ability of the thi6 yeast assay to identify high thiamin lines.
**Fig. S8** Assessing additional considerations for yeast assays: ability to detect HMP‐PP and implications of sample dilution.
**Fig. S9** Comparison of total pyrimidine levels in genetically engineered Arabidopsis lines, measured via yeast assays vs liquid chromatography–tandem mass spectrometry (LC–MS/MS).
**Fig. S10** Comparison of total thiazole levels in genetically engineered Arabidopsis lines, measured via yeast assays vs liquid chromatography–tandem mass spectrometry (LC–MS/MS).
**Fig. S11** Preliminary results of utilizing thi4 yeast assay to estimate sum of total vitamin B1 (vitB1) and thiazole content in brown and polished rice seeds.
**Methods S1** Yeast strains.
**Methods S2** Sample preparation.
**Methods S3** Vitamin B1 (VitB1) standards.
**Methods S4** Yeast cultures.
**Methods S5** Assay protocol, conditions and data acquisition.
**Methods S6** Spiking of plant extracts.
**Table S1** Overview of the different yeast lines.
**Table S2** Identification of spiked metabolite in plant samples by using a scoring grid table.
Please note: Wiley Blackwell are not responsible for the content or functionality of any Supporting Information supplied by the authors. Any queries (other than missing material) should be directed to the *New Phytologist* Central Office.

## Data Availability

The data that support the findings of this study are available from the corresponding author upon reasonable request.

## References

[nph17974-bib-0001] Ajjawi I , Rodriguez Milla MA , Cushman J , Shintani DK . 2007a. Thiamin pyrophosphokinase is required for thiamin cofactor activation in Arabidopsis. Plant Molecular Biology 65: 151–162. 17611796 10.1007/s11103-007-9205-4

[nph17974-bib-0002] Ajjawi I , Tsegaye Y , Shintani D . 2007b. Determination of the genetic, molecular, and biochemical basis of the *Arabidopsis thaliana* thiamin auxotroph *th1* . Archives of Biochemistry and Biophysics 459: 107–114. 17174261 10.1016/j.abb.2006.11.011

[nph17974-bib-0005] Blancquaert D , Storozhenko S , Van Daele J , Stove C , Visser RGF , Lambert W , Van Der Straeten D . 2013. Enhancing pterin and para‐aminobenzoate content is not sufficient to successfully biofortify potato tubers and *Arabidopsis thaliana* plants with folate. Journal of Experimental Botany 64: 3899–3909. 23956417 10.1093/jxb/ert224

[nph17974-bib-0006] Bocobza SE , Malitsky S , Araujo WL , Nunes‐Nesi A , Meir S , Shapira M , Fernie AR , Aharoni A . 2013. Orchestration of thiamin biosynthesis and central metabolism by combined action of the thiamin pyrophosphate riboswitch and the circadian clock in Arabidopsis. Plant Cell 25: 288–307. 23341335 10.1105/tpc.112.106385 PMC3584542

[nph17974-bib-0007] Bouis HE , Saltzman A . 2017. Improving nutrition through biofortification: a review of HarvestPlus, 2003 through 2016. Global Food Security 12: 49–58. 28580239 10.1016/j.gfs.2017.01.009 PMC5439484

[nph17974-bib-0008] Chatterjee A , Abeydeera ND , Bale S , Pai PJ , Dorrestein PC , Russell DH , Ealick SE , Begley TP . 2011. *Saccharomyces cerevisiae* THI4p is a suicide thiamine thiazole synthase. Nature 478: 542–U146. 22031445 10.1038/nature10503 PMC3205460

[nph17974-bib-0009] Coquille S , Roux C , Fitzpatrick TB , Thore S . 2012. The last piece in the vitamin B1 biosynthesis puzzle: structural and functional insight into yeast 4‐amino‐5‐hydroxymethyl‐2‐methylpyrimidine phosphate (HMP‐P) synthase. Journal of Biological Chemistry 287: 42333–42343. 23048037 10.1074/jbc.M112.397240 PMC3516776

[nph17974-bib-0010] Dinicolantonio JJ , Liu J , O'Keefe JH . 2018. Thiamine and cardiovascular disease: a literature review. Progress in Cardiovascular Diseases 61: 27–32. 29360523 10.1016/j.pcad.2018.01.009

[nph17974-bib-0011] Dong W , Stockwell VO , Goyer A . 2015. Enhancement of thiamin content in *Arabidopsis thaliana* by metabolic engineering. Plant and Cell Physiology 56: 2285–2296. 26454882 10.1093/pcp/pcv148

[nph17974-bib-0012] Dong W , Thomas N , Ronald PC , Goyer A . 2016. Overexpression of thiamin biosynthesis genes in rice increases leaf and unpolished grain thiamin content but not resistance to *Xanthomonas* *oryzae* *pv*. *oryzae* . Frontiers in Plant Science 7: 616. 27242822 10.3389/fpls.2016.00616 PMC4861732

[nph17974-bib-0013] Edwards KA , Tu‐Maung N , Cheng K , Wang B , Baeumner AJ , Kraft CE . 2017. Thiamine assays‐advances, challenges, and caveats. ChemistryOpen 6: 178–191. 28413748 10.1002/open.201600160 PMC5390807

[nph17974-bib-0014] Farre G , Blancquaert D , Capell T , Van Der Straeten D , Christou P , Zhu CF . 2014. Engineering complex metabolic pathways in plants. Annual Review of Plant Biology 65: 187–223. 10.1146/annurev-arplant-050213-035825 24579989

[nph17974-bib-0015] Fitzpatrick TB , Chapman LM . 2020. The importance of thiamine (vitamin B‐1) in plant health: from crop yield to biofortification. Journal of Biological Chemistry 295: 12002–12013. 32554808 10.1074/jbc.REV120.010918 PMC7443482

[nph17974-bib-0016] Garg M , Sharma N , Sharma S , Kapoor P , Kumar A , Chunduri V , Arora P . 2018. Biofortified crops generated by breeding, agronomy, and transgenic approaches are improving lives of millions of people around the world. Frontiers in Nutrition 5: 12. 29492405 10.3389/fnut.2018.00012 PMC5817065

[nph17974-bib-0017] Goyer A . 2017. Thiamin biofortification of crops. Current Opinion in Biotechnology 44: 1–7. 27750185 10.1016/j.copbio.2016.09.005

[nph17974-bib-0018] Goyer A , Sweek K . 2011. Genetic diversity of thiamin and folate in primitive cultivated and wild potato (*Solanum*) species. Journal of Agricultural and Food Chemistry 59: 13072–13080. 22088125 10.1021/jf203736e

[nph17974-bib-0019] Hsieh WY , Liao JC , Wang HT , Hung TH , Tseng CC , Chung TY , Hsieh MH . 2017. The Arabidopsis thiamin‐deficient mutant pale green1 lacks thiamin monophosphate phosphatase of the vitamin B‐1 biosynthesis pathway. The Plant Journal 91: 145–157. 28346710 10.1111/tpj.13552

[nph17974-bib-0020] Jiang L , Strobbe S , Van Der Straeten D , Zhang C . 2021. Regulation of plant vitamin metabolism: backbone of biofortification for the alleviation of hidden hunger. Molecular Plant 14: 40–60. 33271336 10.1016/j.molp.2020.11.019

[nph17974-bib-0021] Kall MA . 2003. Determination of total vitamin B‐6 in foods by isocratic HPLC: a comparison with microbiological analysis. Food Chemistry 82: 315–327.

[nph17974-bib-0022] Kamarudin AN , Lai KS , Lamasudin DU , Idris AS , Yusof ZNB . 2017. Enhancement of thiamine biosynthesis in oil palm seedlings by colonization of endophytic fungus *Hendersonia* *toruloidea* . Frontiers in Plant Science 8: 1799. 29089959 10.3389/fpls.2017.01799 PMC5651052

[nph17974-bib-0023] Kartal B , Palabiyik B . 2019. Thiamine leads to oxidative stress resistance via regulation of the glucose metabolism. Cellular and Molecular Biology 65: 73–77. 30782298

[nph17974-bib-0024] Kawasaki Y , Onozuka M , Mizote T , Nosaka K . 2005. Biosynthesis of hydroxymethylpyrimidine pyrophosphate in *Saccharomyces cerevisiae* . Current Genetics 47: 156–162. 15614489 10.1007/s00294-004-0557-x

[nph17974-bib-0025] Li J , Liu J , Wen W , Zhang P , Wan Y , Xia X , Zhang Y , He Z . 2018. Genome‐wide association mapping of vitamins B1 and B2 in common wheat. Crop Journal 6: 263–270.

[nph17974-bib-0026] Li KT , Moulin M , Mangel N , Albersen M , Verhoeven‐Duif NM , Ma QX , Zhang P , Fitzpatrick TB , Gruissem W , Vanderschuren H . 2015. Increased bioavailable vitamin B‐6 in field‐grown transgenic cassava for dietary sufficiency. Nature Biotechnology 33: 1029–1032. 10.1038/nbt.3318 26448082

[nph17974-bib-0027] Llorente B , Fairhead C , Dujon B . 1999. Genetic redundancy and gene fusion in the genome of the baker's yeast *Saccharomyces cerevisiae*: functional characterization of a three‐member gene family involved in the thiamine biosynthetic pathway. Molecular Microbiology 32: 1140–1152. 10383756 10.1046/j.1365-2958.1999.01412.x

[nph17974-bib-0028] Lonsdale D . 2006. Review of the biochemistry, metabolism and clinical benefits of thiamin(e) and its derivatives. Evidence‐Based Complementary and Alternative Medicine 3: 49–59. 16550223 10.1093/ecam/nek009 PMC1375232

[nph17974-bib-0029] Mangel N . 2016. Natural variation, molecular determinants and genetic engineering of vitamin B1 and vitamin B6 biosynthesis in cassava and rice, PhD thesis. Zurich, Switzerland: ETH Zurich, Germany.

[nph17974-bib-0030] Mangel N , Fudge JB , Fitzpatrick TB , Gruissem W , Vanderschuren H . 2017. Vitamin B‐1 diversity and characterization of biosynthesis genes in cassava. Journal of Experimental Botany 68: 3351–3363. 28859374 10.1093/jxb/erx196 PMC5853225

[nph17974-bib-0031] Mangel N , Fudge JB , Li KT , Wu TY , Tohge T , Fernie AR , Szurek B , Fitzpatrick TB , Gruissem W , Vanderschuren H . 2019. Enhancement of vitamin B‐6 levels in rice expressing Arabidopsis vitamin B‐6 biosynthesis *de novo* genes. The Plant Journal 99: 1047–1065. 31063672 10.1111/tpj.14379 PMC6852651

[nph17974-bib-0032] Martinis J , Gas‐Pascual E , Szydlowski N , Crevecoeur M , Gisler A , Burkle L , Fitzpatrick TB . 2016. Long‐distance transport of thiamine (vitamin B1) is concomitant with that of polyamines. Plant Physiology 171: 542–553. 27006489 10.1104/pp.16.00009 PMC4854701

[nph17974-bib-0033] Mimura M , Zallot R , Niehaus TD , Hasnain G , Gidda SK , Nguyen TND , Anderson EM , Mullen RT , Brown G , Yakunin AF *et al*. 2016. Arabidopsis *TH2* encodes the orphan enzyme thiamin monophosphate phosphatase. Plant Cell 28: 2683–2696. 27677881 10.1105/tpc.16.00600 PMC5134987

[nph17974-bib-0034] Moulin M , Nguyen GT , Scaife MA , Smith AG , Fitzpatrick TB . 2013. Analysis of Chlamydomonas thiamin metabolism *in vivo* reveals riboswitch plasticity. Proceedings of the National Academy of Sciences, USA 110: 14622–14627. 10.1073/pnas.1307741110 PMC3767531 23959877

[nph17974-bib-0035] Murashige T , Skoog F . 1962. A revised medium for rapid growth and bio assays with tobacco tissue cultures. Physiologia Plantarum 15: 473–497.

[nph17974-bib-0036] Nishimura H , Kawasaki Y , Nosaka K , Kaneko Y , Iwashima A . 1991. A constitutive thiamine metabolism mutation, *thi80*, causing reduced thiamine pyrophosphokinase activity in *Saccharomyces cerevisiae* . Journal of Bacteriology 173: 2716–2719. 1849514 10.1128/jb.173.8.2716-2719.1991 PMC207844

[nph17974-bib-0037] Niven CF , Smiley KL . 1943. A microbiological assay method for thiamine. Journal of Biological Chemistry 150: 1–9.

[nph17974-bib-0038] Noordally ZB , Trichtinger C , Dalvit I , Hofmann M , Roux C , Zamboni N , Pourcel L , Gas‐Pascual E , Gisler A , Fitzpatrick TB . 2020. The coenzyme thiamine diphosphate displays a daily rhythm in the Arabidopsis nucleus. Communications Biology 3: 1–13. 32372067 10.1038/s42003-020-0927-z PMC7200797

[nph17974-bib-0039] Nosaka K . 2006. Recent progress in understanding thiamin biosynthesis and its genetic regulation in *Saccharomyces cerevisiae* . Applied Microbiology and Biotechnology 72: 30–40. 16826377 10.1007/s00253-006-0464-9

[nph17974-bib-0040] Nosaka K , Kaneko Y , Nishimura H , Iwashima A . 1993. Isolation and characterization of a thiamin pyrophosphokinase gene, *THI80*, from *Saccharomyces cerevisiae* . Journal of Biological Chemistry 268: 17440–17447. 8394343

[nph17974-bib-0041] Nosaka K , Nishimura H , Kawasaki Y , Tsujihara T , Iwashima A . 1994. Isolation and characterization of the *THI6* gene encoding a bifunctional thiamin‐phosphate pyrophosphorylase hydroxyethylthiazole kinase from *Saccharomyces cerevisiae* . Journal of Biological Chemistry 269: 30510–30516. 7982968

[nph17974-bib-0042] Nosaka K , Onozuka M , Kakazu N , Hibi S , Nishimura H , Nishino H , Abe T . 2001. Isolation and characterization of a human thiamine pyrophosphokinase cDNA. Biochimica et Biophysica Acta‐Gene Structure and Expression 1517: 293–297. 10.1016/s0167-4781(00)00247-5 11342111

[nph17974-bib-0043] Pourcel L , Moulin M , Fitzpatrick TB . 2013. Examining strategies to facilitate vitamin B1 biofortification of plants by genetic engineering. Frontiers in Plant Sciences 4: 160. 10.3389/fpls.2013.00160 PMC3665906 23755056

[nph17974-bib-0044] Rao VLR , Butterworth RF . 1995. Thiamine phosphatases in human brain ‐ Regional alterations in patients with alcoholic cirrhosis. Alcoholism‐Clinical and Experimental Research 19: 523–526. 7625592 10.1111/j.1530-0277.1995.tb01541.x

[nph17974-bib-0045] Raschke M , Boycheva S , Crevecoeur M , Nunes‐Nesi A , Witt S , Fernie AR , Amrhein N , Fitzpatrick TB . 2011. Enhanced levels of vitamin B‐6 increase aerial organ size and positively affect stress tolerance in Arabidopsis. The Plant Journal 66: 414–432. 21241390 10.1111/j.1365-313X.2011.04499.x

[nph17974-bib-0046] Raschke M , Burkle L , Muller N , Nunes‐Nesi A , Fernie AR , Arigoni D , Amrhein N , Fitzpatrick TB . 2007. Vitamin B1 biosynthesis in plants requires the essential iron‐sulfur cluster protein, THIC. Proceedings of the National Academy of Sciences, USA 104: 19637–19642. 10.1073/pnas.0709597104 PMC2148341 18048325

[nph17974-bib-0047] Saltzman A , Birol E , Bouis HE , Boy E , De Moura FF , Islam Y , Pfeiffer WH . 2013. Biofortification: progress toward a more nourishing future. Global Food Security‐Agriculture Policy Economics and Environment 2: 9–17.

[nph17974-bib-0048] Schaub P , Al‐Babili S , Drake R , Beyer P . 2005. Why is golden rice golden (yellow) instead of red? Plant Physiology 138: 441–450. 15821145 10.1104/pp.104.057927 PMC1104197

[nph17974-bib-0049] Schultz A , Atkin L , Frey C . 1942. Determination of vitamin B1 by yeast fermentation method improvements related to use of sulfite cleavage and a new fermentometer. Industrial & Engineering Chemistry Analytical Edition 14: 35–39.

[nph17974-bib-0050] Strobbe S , Van Der Straeten D . 2018. Toward eradication of B‐vitamin deficiencies: considerations for crop biofortification. Frontiers in Plant Science 9: 443. 29681913 10.3389/fpls.2018.00443 PMC5897740

[nph17974-bib-0051] Strobbe S , Verstraete J , Stove C , Van Der Straeten D . 2021a. Metabolic engineering of rice endosperm towards higher vitamin B1 accumulation. Plant Biotechnology Journal 19: 1253–1267. 33448624 10.1111/pbi.13545 PMC8196658

[nph17974-bib-0052] Strobbe S , Verstraete J , Stove C , Van Der Straeten D . 2021b. Metabolic engineering provides insight into the regulation of thiamin biosynthesis in plants. Plant Physiology 186: 1832–1847. 33944954 10.1093/plphys/kiab198 PMC8331165

[nph17974-bib-0053] Tambasco‐Studart M , Titiz O , Raschle T , Forster G , Amrhein N , Fitzpatrick TB . 2005. Vitamin B6 biosynthesis in higher plants. Proceedings of the National Academy of Sciences, USA 102: 13687–13692. 10.1073/pnas.0506228102 PMC1224648 16157873

[nph17974-bib-0054] Titiz O , Tambasco‐Studart M , Warzych E , Apel K , Amrhein N , Laloi C , Fitzpatrick TB . 2006. PDX1 is essential for vitamin B6 biosynthesis, development and stress tolerance in Arabidopsis. The Plant Journal 48: 933–946. 17227548 10.1111/j.1365-313X.2006.02928.x

[nph17974-bib-0055] Trivedi A , Arya L , Verma S , Tyagi R , Hemantaranjan A , Verma M , Sharma V , Saha D . 2018. Molecular profiling of foxtail millet (*Setaria italica (L.) P. Beauv*) from central Himalayan region for genetic variability and nutritional quality. Journal of Agricultural Science 156: 333–341.

[nph17974-bib-0056] Van Der Straeten D , Bhullar NK , De Steur H , Gruissem W , Mackenzie D , Pfeiffer W , Qaim M , Slamet‐Loedin I , Strobbe S , Tohme J *et al*. 2020. Multiplying the efficiency and impact of biofortification through metabolic engineering. Nature Communications 11: 5203. 10.1038/s41467-020-19020-4 PMC7567076 33060603

[nph17974-bib-0057] Verstraete J , Strobbe S , Van Der Straeten D , Stove C . 2020. The first comprehensive LC–MS/MS method allowing dissection of the thiamine pathway in plants. Analytical Chemistry 92: 4073–4081. 32056423 10.1021/acs.analchem.9b05717

[nph17974-bib-0058] Verstraete J , Strobbe S , Van Der Straeten D , Stove C . 2021. An optimized LC‐MS/MS method as a pivotal tool to steer thiamine biofortification strategies in rice. Talanta 224: 121905. 33379109 10.1016/j.talanta.2020.121905

[nph17974-bib-0059] Wang LC , Ye XF , Liu HC , Liu XJ , Wei CC , Huang YQ , Liu YJ , Tu JM . 2016. Both overexpression and suppression of an *Oryza* *sativa* NB‐LRR‐like gene *OsLSR* result in autoactivation of immune response and thiamine accumulation. Scientific Reports 6: 24079. 27052628 10.1038/srep24079 PMC4823736

[nph17974-bib-0060] Whitfield KC , Bourassa MW , Adamolekun B , Bergeron G , Bettendorff L , Brown KH , Cox L , Fattal‐Valevski A , Fischer PR , Frank EL *et al*. 2018. Thiamine deficiency disorders: diagnosis, prevalence, and a roadmap for global control programs. Annals of the New York Academy of Sciences 1430: 3–43. 30151974 10.1111/nyas.13919 PMC6392124

[nph17974-bib-0061] Wightman R , Meacock PA . 2003. The *THI5* gene family of *Saccharomyces cerevisiae*: distribution of homologues among the hemiascomycetes and functional redundancy in the aerobic biosynthesis of thiamin from pyridoxine. Microbiology 149: 1447–1460. 12777485 10.1099/mic.0.26194-0

[nph17974-bib-0062] Wilson RB . 2020. Pathophysiology, prevention, and treatment of beriberi after gastric surgery. Nutrition Reviews 78: 1015–1029. 32388553 10.1093/nutrit/nuaa004 PMC7666909

[nph17974-bib-0063] Yin Y , Tian L , Li X , Huang M , Liu L , Wu P , Li M , Jiang H , Wu G , Chen YJPS . 2019. The role of endogenous thiamine produced via THIC in root nodule symbiosis in *Lotus japonicus* . Plant Science 283: 311–320. 31128701 10.1016/j.plantsci.2019.03.011

