# Supplementary material for: A novel panel of yeast assays for the assessment of thiamin and its biosynthetic intermediates in plant tissues
Source: New Phytol. 2022 Feb 8;234(2):748–63. doi: 10.1111/nph.17974 (PMC9303440; doi:10.1111/nph.17974)
Supplement: Supplementary file 1 — Fig. S1 Schematic overview of the sample preparation procedure for the liquid chromatography–tandem mass spectrometry (LC–MS/MS) method. Fig. S2 Schematic representation of yeast microbiological assays for thiamin determination. Fig. S3 Schematic overview of blinded yeast assays using metabolite‐spiked plant extracts. Fig. S4 Repeat of spiking experiment. Fig. S5 Analysis of 60 different metabolite‐spiked plant extracts with different yeast assays. Fig. S6 Results of thi20/21 yeast assay applied on both Arabidopsis grown on thiamin and genetically engineered lines. Fig. S7 Assessing the ability of the thi6 yeast assay to identify high thiamin lines. Fig. S8 Assessing additional considerations for yeast assays: ability to detect HMP‐PP and implications of sample dilution. Fig. S9 Comparison of total pyrimidine levels in genetically engineered Arabidopsis lines, measured via yeast assays vs liquid chromatography–tandem mass spectrometry (LC–MS/MS). Fig. S10 Comparison of total thiazole levels in genetically engineered Arabidopsis lines, measured via yeast assays vs liquid chromatography–tandem mass spectrometry (LC–MS/MS). Fig. S11 Preliminary results of utilizing thi4 yeast assay to estimate sum of total vitamin B1 (vitB1) and thiazole content in brown and polished rice seeds. Methods S1 Yeast strains. Methods S2 Sample preparation. Methods S3 Vitamin B1 (VitB1) standards. Methods S4 Yeast cultures. Methods S5 Assay protocol, conditions and data acquisition. Methods S6 Spiking of plant extracts. Table S1 Overview of the different yeast lines. Table S2 Identification of spiked metabolite in plant samples by using a scoring grid table. Please note: Wiley Blackwell are not responsible for the content or functionality of any Supporting Information supplied by the authors. Any queries (other than missing material) should be directed to the New Phytologist Central Office. [file NPH-234-748-s001.pdf]

New Phytologist

New Phytologist **Supporting Information**

Article title: **A novel panel of yeast assays for the assessment of thiamin and its biosynthetic intermediates in plant tissues**

Authors: Simon Strobbe, Jana Verstraete, Teresa B. Fitzpatrick, Maria Faustino, Tiago F. Lourenço, M. Margarida Oliveira, Christophe Stove and Dominique Van Der Straeten

Article acceptance date: 05 January 2022

The following Supporting Information is available for this article:

Fig. S1 Schematic overview of the sample preparation procedure for the LC-MS/MS method

Fig. S2 Schematic representation of yeast microbiological assays for thiamin determination**.**

Fig. S3 Schematic overview of blinded yeast assays using metabolite-spiked plant extracts.

Fig. S4 Repeat of spiking experiment.

Fig. S5 Analysis of 60 different metabolite-spiked plant extracts with different yeast assays.

Fig. S6 Results of *thi20/21* yeast assay applied on both Arabidopsis grown on thiamin and genetically engineered lines.

Fig. S7 Assessing the ability of the *thi6* yeast assay to identify high thiamin lines.

Fig. S8 Assessing additional considerations for yeast assays: ability to detect HMP-PP and implications of sample dilution.

Fig. S9 Comparison of total pyrimidine levels in genetically engineered Arabidopsis lines, measured via yeast assays vs. LC-MS/MS.

Fig. S10 Comparison of total thiazole levels in genetically engineered Arabidopsis lines, measured via yeast assays vs. LC-MS/MS.

Fig. S11 Preliminary results of utilizing *thi4* yeast assay to estimate sum of total vitB1 and thiazole content in brown and polished rice seeds

Table S1 Overview of the different yeast lines.

Table S2 Identification of spiked metabolite in plant samples by using a scoring grid table.

Methods S1 Yeast strains

Methods S2 Sample preparation

Methods S3 VitB1 standards

Methods S4 Yeast cultures

Methods S5 Assay protocol, conditions and data acquisition

Methods S6 Spiking of plant extracts

Fig. S1

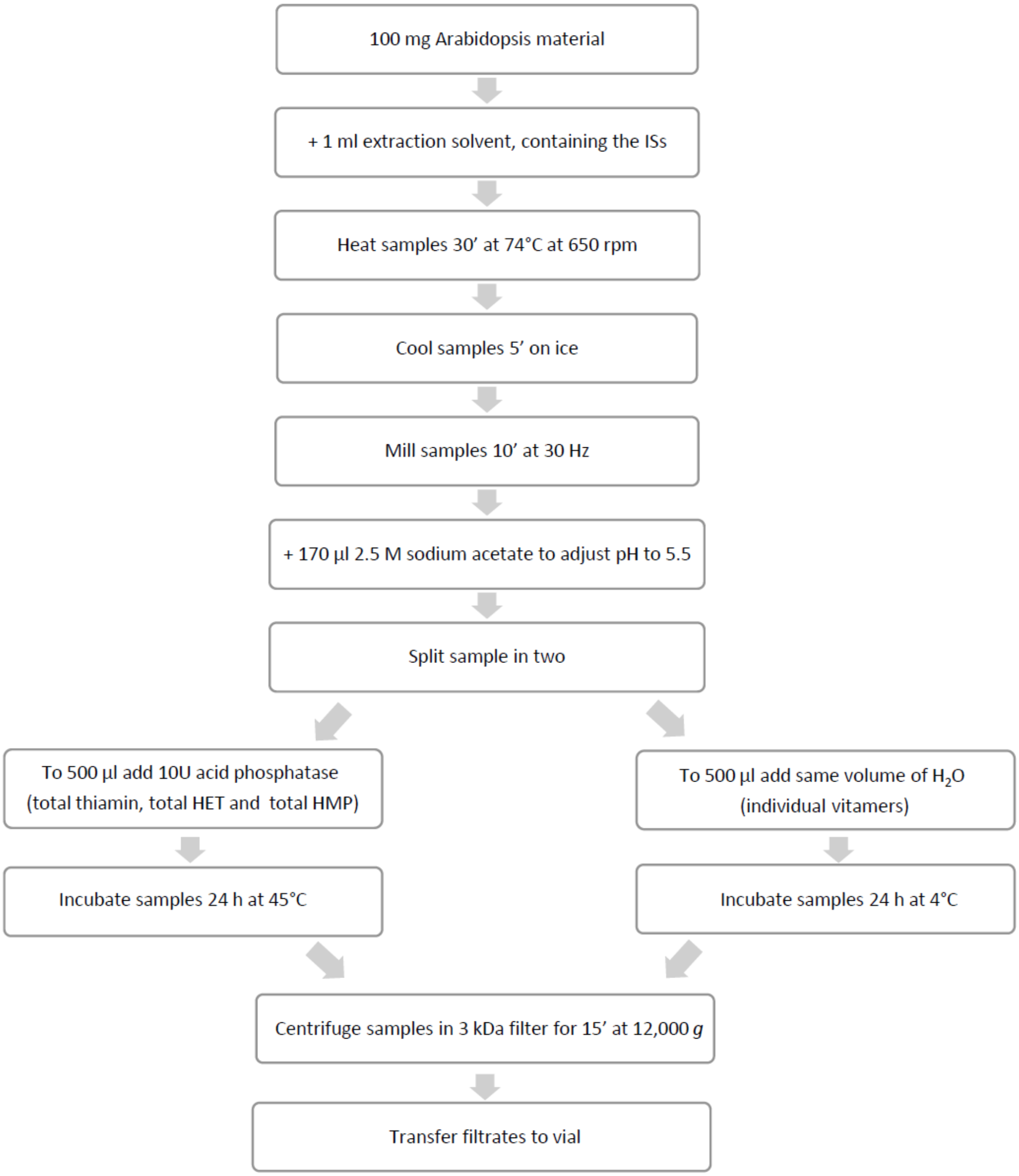

Supporting Fig. S1. Schematic overview of the sample preparation procedure for the LC-MS/MS method. LC-MS/MS determination of thiamin, the precursors HMP and HET, and the phosphate derivatives TMP and TPP in Arabidopsis thaliana, as described in detail by (Verstraete et al., 2020).

Fig. S2

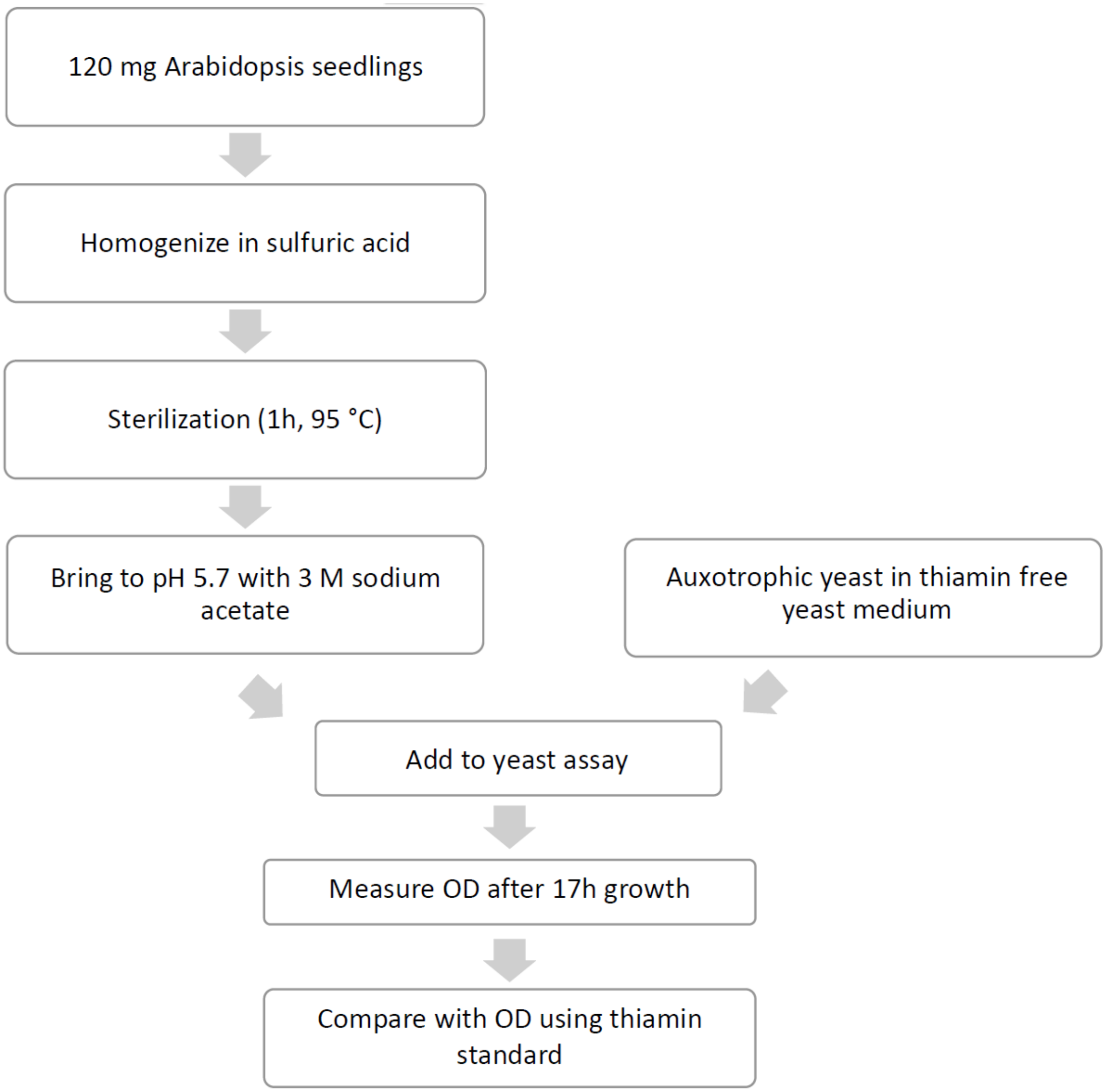

Supporting Fig. S2. Schematic representation of yeast microbiological assays for thiamin determination. The microbiological assay protocol was adapted from the assay utilizing the *Saccharomyces cerevisiae* thiazole biosynthesis mutant, *thi4* (Kall, 2003;Raschke et al., 2007;Chatterjee et al., 2011;Mangel et al., 2017). Abbreviation: OD, optical density.

Fig. S3

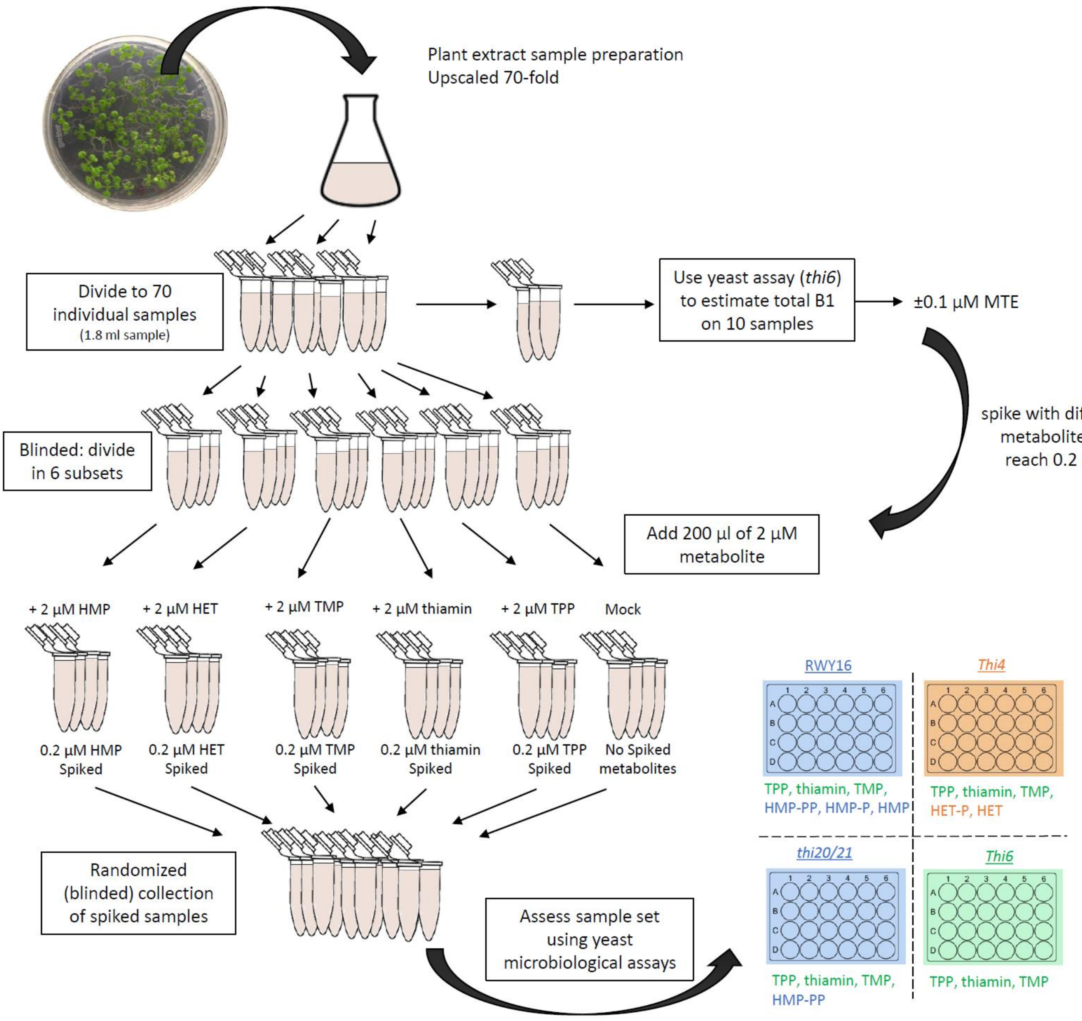

Supporting Fig. S3. Schematic overview of blinded yeast assays using metabolite-spiked plant extracts. Plant extracts are spiked with 2 µM metabolite concentration to reach a final concentration of 0.2 µM in the 2 ml sample. Given that untreated samples contain a molar thiamin equivalent (MTE) of ± 0.1 µM, the final MTE should reach ± 0.3 µM, 3-fold exceeding the non-spiked samples. The four different yeast assays, represented by 24-well plates, are represented and rescuing (measured) metabolites are listed below. The color code is similar to Fig. 1, in which the intermediates of the pyrimidine branch are shown in blue, the intermediates of the thiazole branch in orange and VitB1 metabolites in green.

Fig. S4

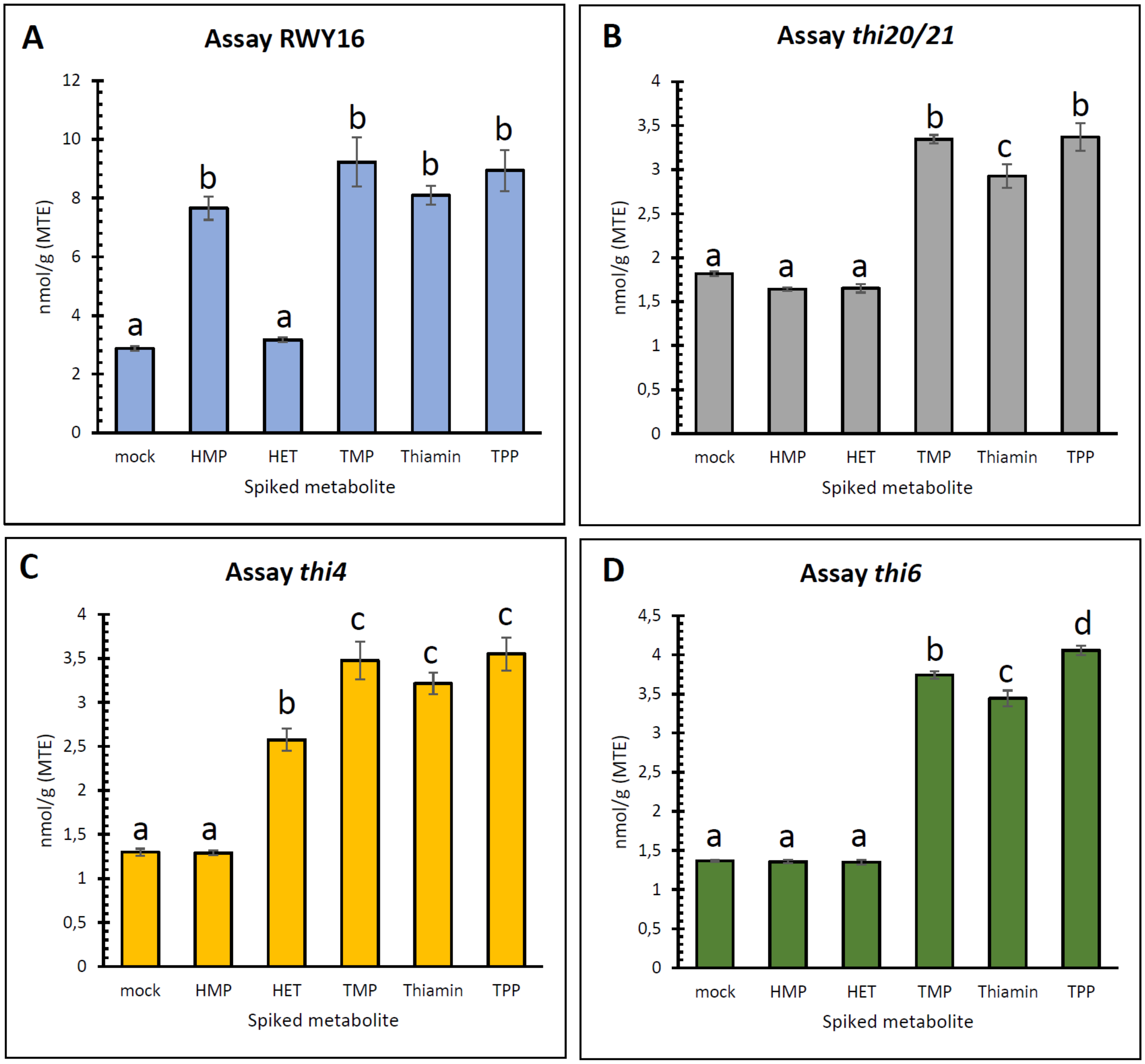

<u>Supporting Fig. S4. Repeat of spiking experiment</u>. Effects of spiking a particular metabolite on the results of the different yeast assays as analyzed in Fig.4 is repeated (not-blinded) using separately diluted batches of metabolites. This was performed to assess whether some discrepancies in measured MTE (molar thiamin equivalent) from the different metabolites can in part be explained by a specific metabolite stock solution. The measured MTE values upon spiking with the different thiamin-related metabolites are depicted for yeast assay RWY16 (**a**; can be rescued by HMP(-P(P)), TMP, thiamin and TPP)), thi20/21 (**b;** can be rescued by HMP-PP, TMP, thiamin and TPP), thi4 (**c;** can be rescued by HET(-P), TMP, thiamin and TPP) and thi6 (**d;** can be rescued by TMP, thiamin and TPP). Bars indicate the mean ± SE of 5 samples, whether or not spiked with a particular metabolite. Significant differences were

determined via parametric tests, as using the Shapiro-Wilk test, the experimental data were found to follow a normal distribution. ANOVA test revealed presence of significant differences within the groups, identified using the post-hoc Tukey's test. Different lower case letters indicate significant differences between groups ($p < 0.05$).

Fig. S5

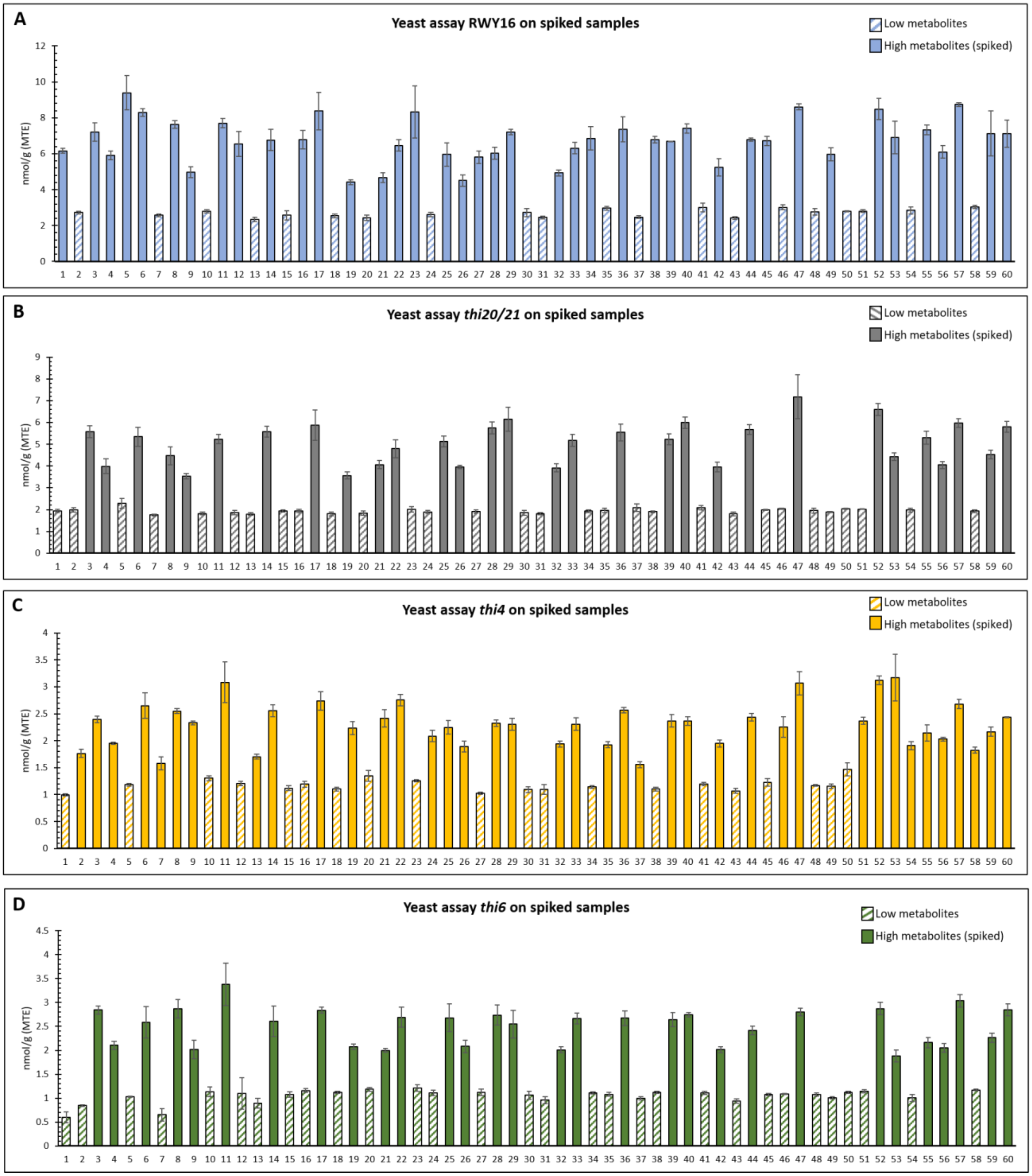

Supporting Fig. S5. Analysis of 60 different metabolite-spiked plant extracts with different yeast assays. Plant samples, originating from 15 days old WT Arabidopsis complete seedlings, grown on thiamin-free half strength MS medium were used as starting material for the analyses. Sample extracts were spiked with HMP, HET, TMP, thiamin

or TPP to test the ability of the yeast assays to identify the increase in a specific thiamin-related metabolite (see schematic representation of the experimental setup in Fig. S3). The molar concentration of the metabolites spiked was set at a specific level, aimed at surmounting the molar thiamin equivalent (MTE) of non-spiked plant samples three-fold. Data represent the mean ± SE of three technical replicates. Samples were defined as harboring an either low (shaded bars) or high metabolite content (full bars). **(a)** Results of screening of 60 samples for an increase in total B1 + pyrimidine by the RWY16 yeast assay. **(b)** Results of screening of 60 samples for an increase in total B1 (+ pyrimidine pyrophosphate) by the *thi20/21* yeast assay. **(c)** Results of screening of 60 samples for an increase in total B1 + thiazole by the *thi4* yeast assay. **(d)** Results of screening of 60 samples for an increase in total B1 by the *thi6* yeast assay.

Fig. S6

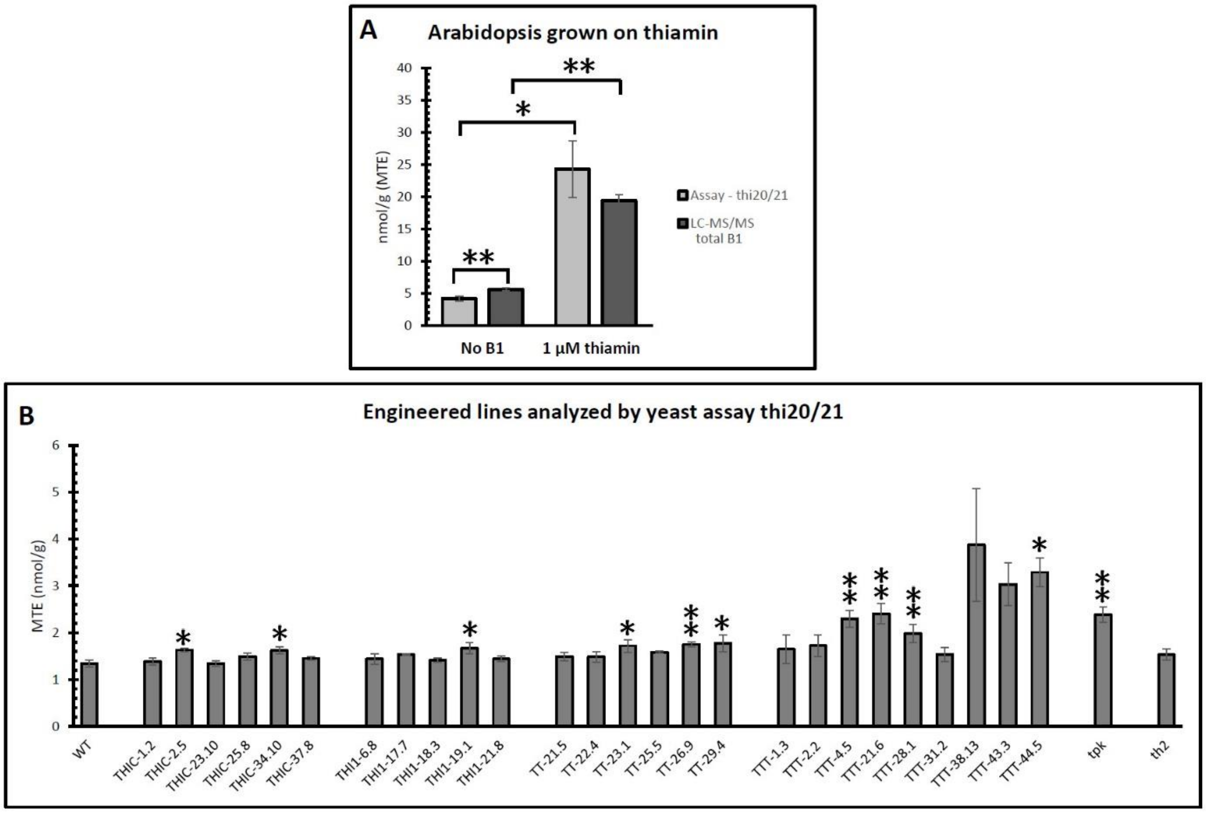

Supporting Fig. S6. Results of *thi20/21* yeast assay applied on both Arabidopsis grown on thiamin and genetically engineered lines. **(a)** Leaf material of Arabidopsis plants, grown on half strength MS medium with or without 1 µM of thiamin for 35 days, was used to test the ability of the *thi20/21* yeast assay to pick up *in planta* differences in thiamin-related metabolites. Based on the genetic knowledge of *Saccharomyces cerevisiae* biosynthesis presented in Figure 1, utilizing strain *thi20/21* is considered to allow the estimation of total B1 as well as HMP pyrophosphate (HMP-PP). Data (both microbiological and LC-MS/MS) are represented as nmol/g plant material, which is further specified as MTE for the microbiological assays. The mean values ± standard error of 5 biological replicates are shown. **(b)** The combined levels of HMP-PP and B1 (MTE, molar thiamin equivalent), as measured by *thi20/21* yeast assay are shown in the examined engineered lines. The engineered lines overexpressing *AtTHIC* (THIC), *AtTHI1* (THI1), both *AtTHIC* and *AtTHI1* (TT) or *AtTHIC*, *AtTHI1* and *AtTH1* (TTT), described by (Strobbe et al., 2021b), were utilized. The datasets (in A and B) were tested for normality using the Shapiro-Wilk test. In case of normality, statistically significant differences were detected via a two-sided T-test (scedasticity depending on the outcome of a

preceding F-test). In case of non-normality, Mann-Whitney U test was used to identify significant differences. Significant differences are indicated by a single asterisk ($p < 0.05$) or double asterisks ($p < 0.01$). For the engineered lines (B), the significant difference depict comparison with the wild type (WT).

Fig. S7

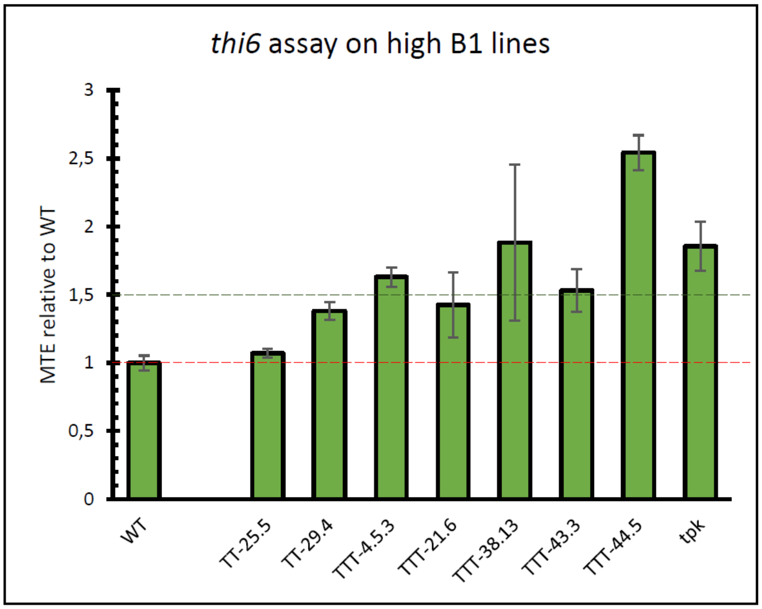

<u>Supporting Fig. S7. Assessing the ability of the *thi6* yeast assay to identify high thiamin lines.</u> Here, MTE values acquired via *thi6* yeast assay of the lines, harboring above 1.5-fold increase in total vitB1 level, as measured by LC-MS/MS and compared to WT, are presented. The engineered lines overexpressing *AtTHIC* (THIC), *AtTHI1* (THI1), both *AtTHIC* and *AtTHI1* (TT) or *AtTHIC*, *AtTHI1* and *AtTH1* (TTT), described by (Strobbe et al., 2021b), were utilized. Eight engineered lines depicted above 1.5-fold enhancement of total B1 levels, when measured by LC-MS/MS analysis (Fig. 6a). Mean values ± SE of 3 (transgenic) or 6 (WT) biological replicates are shown. This graph zooms in on the result of the *thi6* yeast assay of these lines, to examined whether this above 1.5-fold enrichment in *in planta* vitB1 level is recognized by the *thi6* assay. These lines are indeed found to exhibit MTE values above WT, albeit very limited for line TT-25.5 (WT level is set to 1, red dashed line). The black dashed line corresponds to the 1.5-fold increase of MTE over WT, which is the threshold level needed to be reached by LC-MS/MS metabolite level to be selected here. Given the presented correlation between the LC-MS/MS and *thi6* assay data (relative values; see Fig. 6), the number of false negatives, upon utilization of the *thi6* assay as a screening method, is expected to be low. Abbreviations: MTE, molar thiamin equivalent; WT, wild type.

Fig. S8

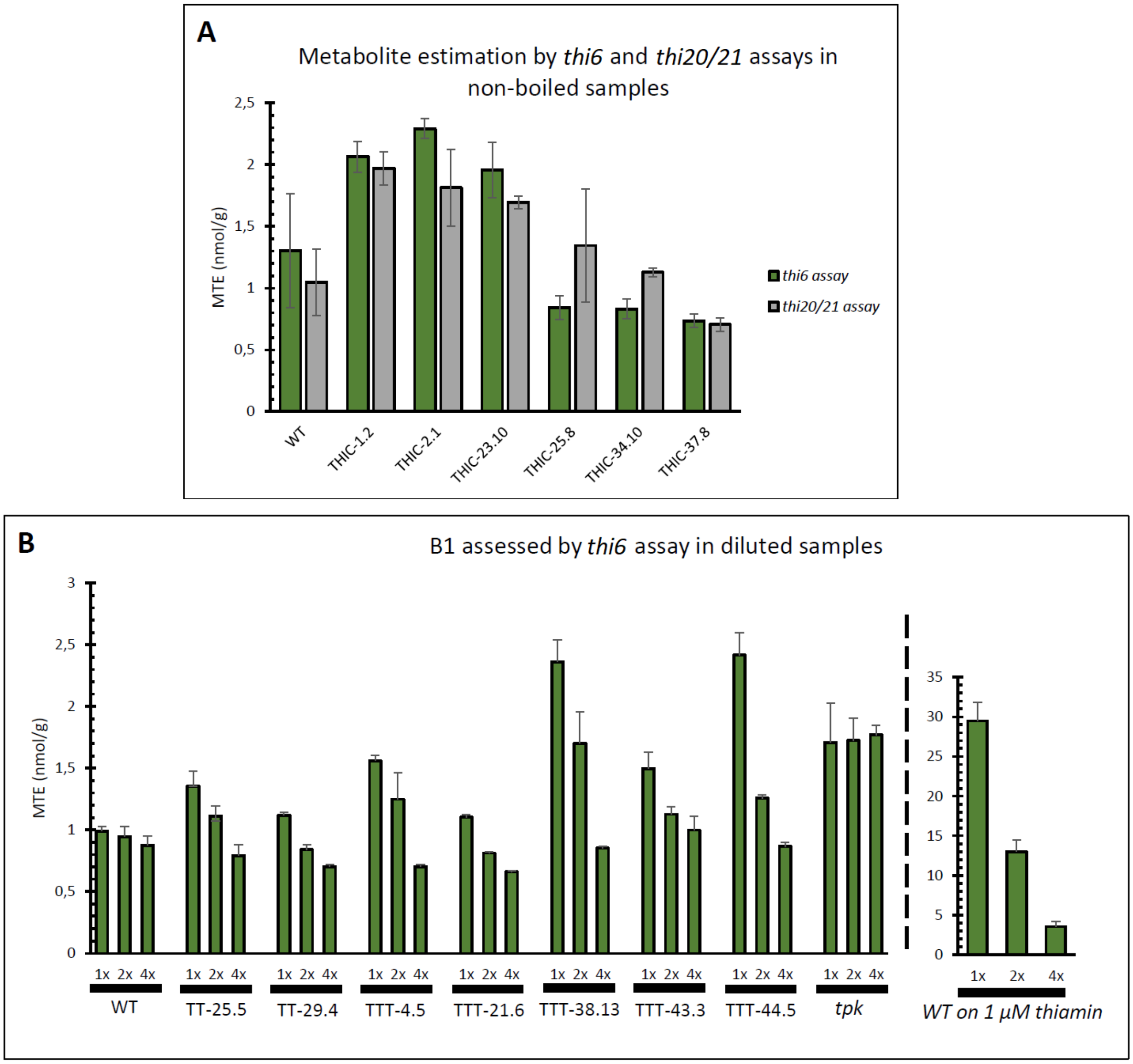

Supporting Fig. S8. Assessing additional considerations for yeast assays: ability to detect HMP-PP and implications of sample dilution. The engineered lines overexpressing *AtTHIC* (THIC), *AtTHI1* (THI1), both *AtTHIC* and *AtTHI1* (TT) or *AtTHIC*, *AtTHI1* and *AtTH1* (TTT), described by (Strobbe et al., 2021b), were utilized. **(a)** As the only difference in the set of metabolites detected by the *THI6* and *THI20/21* assays is that the latter is theoretically also detecting the pyrimidine intermediate HMP-PP (Fig. 1b), their combined usage could be considered a crude method of estimating HMP-PP presence in samples. Unfortunately, this pyrophosporylated entity cannot be detected in the LC-MS/MS method (as it depends on measurement of phosphatase treated samples, thereby not enabling the

distinction between HMP-P and HMP-PP) (Verstraete et al., 2020), making it impossible to verify the relative presence of this metabolite. As HMP-PP is a labile compound, it could have deteriorated in the cooking step (sterilization of plant samples). Therefore, *thi6* and *thi20/21* assays were conducted on samples of transgenic plants (selected to have a high probability of HMP-PP presence, due to high pyrimidine accumulation), which were filter sterilized (no cooking step was included in sample preparation).The higher MTE measured in the *thi20/21* assay as compared to the *thi6* assay observed in line THIC-34.10 (and THIC-25.8, though less clear), could hint to a potential effect of HMP-PP content, yet this effect is not sufficiently clear to allow such conclusion. Values depict means of 4 technical replicates (transgenic lines) or 3 biological replicates (WT) ± SE. **(b)** To assess the concentration range within which relative metabolite measurements can made, dilution of samples were tested using the *thi6* assay. The original concentration was tested, as well as a 2-fold (2x) and 4-fold dilution (4x). Values show mean ±SE of different dilution measured by 4 technical replicates. Abbreviations: MTE, molar thiamin equivalent; WT, wild type.

Fig. S9

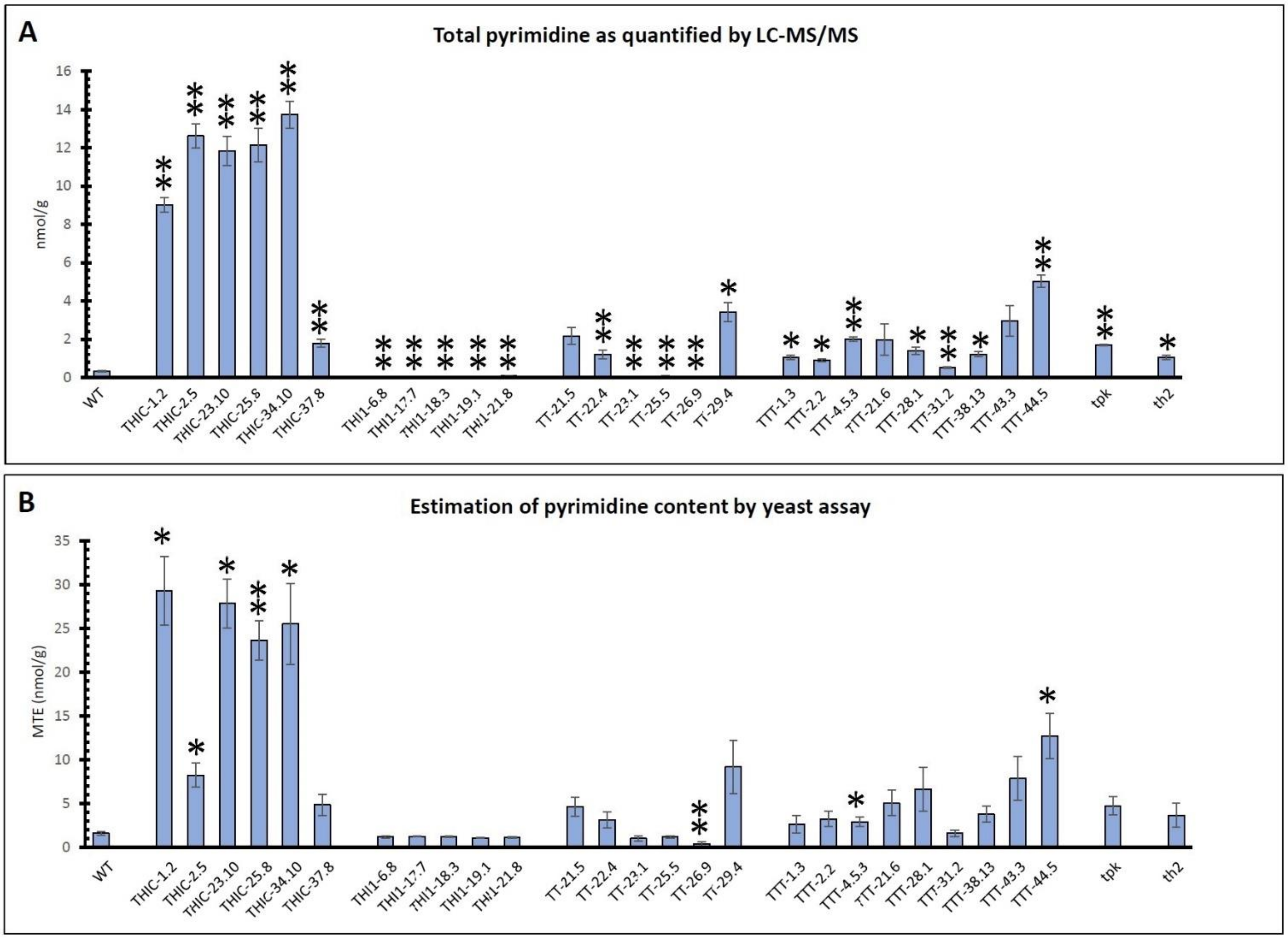

<u>Supporting Fig. S9. Comparison of total pyrimidine levels in genetically engineered Arabidopsis lines, measured via yeast assays vs. LC-MS/MS.</u> The engineered lines overexpressing *AtTHIC* (THIC), *AtTHI1* (THI1), both *AtTHIC* and *AtTHI1* (TT) or *AtTHIC*, *AtTHI1* and *AtTH1* (TTT), described by (Strobbe et al., 2021b), were utilized. To obtain an estimate of the pyrimidine intermediate accumulation in genetically engineered Arabidopsis plants, results from yeast assays using RWY16 and *thi6* were compared. Differences between both yeast assays (RWY16-*thi6*) could be attributed to the accumulation of HMP(-P(P)), the pyrimidine intermediate in thiamin biosynthesis. Mean values ± SE of 3 (transgenic) or 6 (WT) biological replicates are shown. **(a)** Levels of total pyrimidine, as measured by LC-MS/MS after phosphatase treatment, are depicted. **(b)** Differences in MTE from RWY16 and *thi6* yeast assay are shown, serving as an estimate for pyrimidine (HMP-(P(P))) accumulation. The datasets were tested for normality using the Shapiro-Wilk test. In case of normality, statistically significant differences were detected via a two-sided T-test

(scedasticity depending on the outcome of a preceding F-test). In case of non-normality, Mann-Whitney U test was used to identify significant differences. Significant differences are indicated by a single asterisk ($p < 0.05$) or double asterisks ($p < 0.01$). For the engineered lines (B), the significant difference depict comparison with the wild type (WT).

Fig. S10

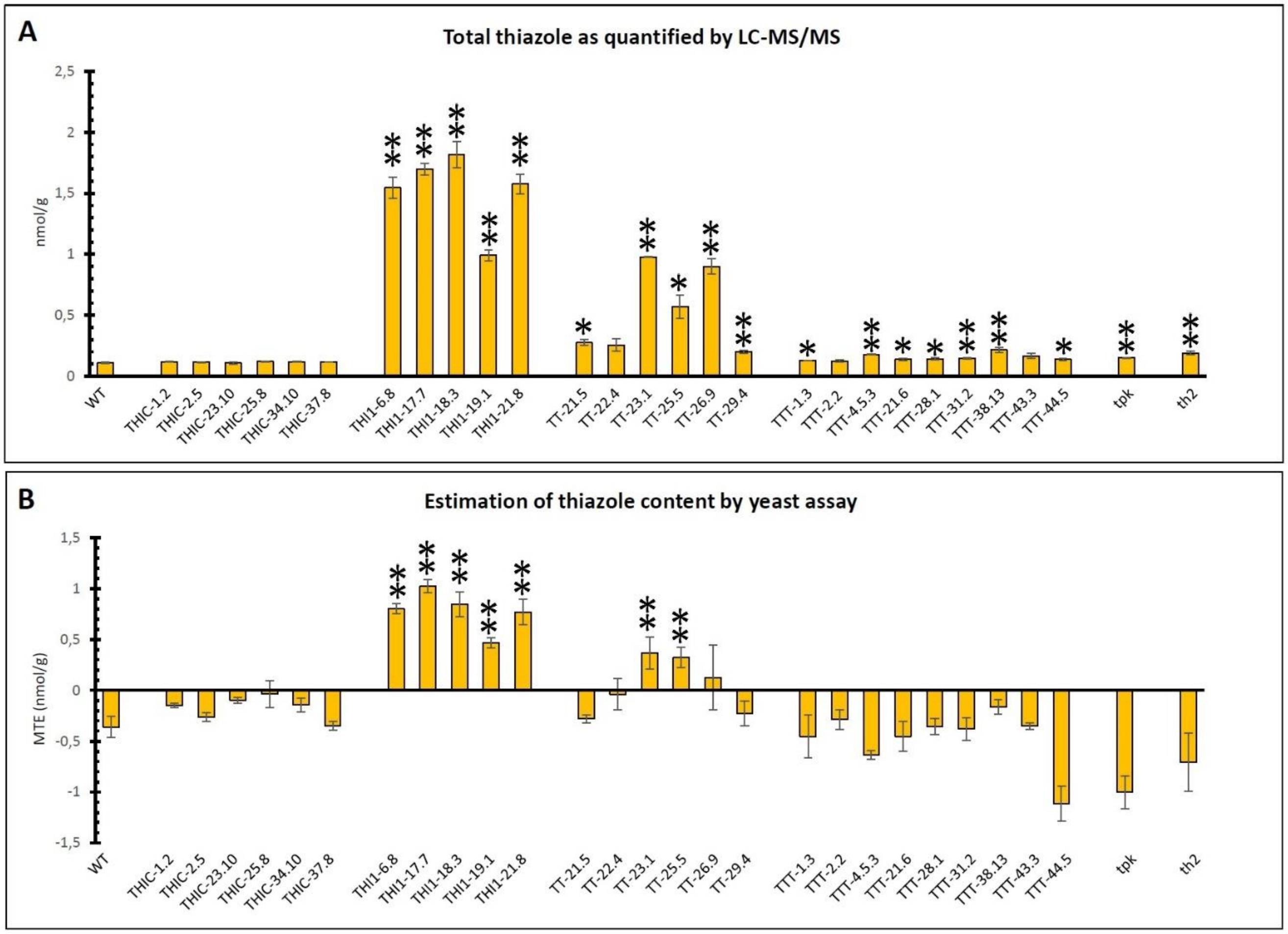

<u>Supporting Fig. S10. Comparison of total thiazole levels in genetically engineered Arabidopsis lines, measured via yeast assays vs. LC-MS/MS.</u> The engineered lines overexpressing *AtTHIC* (THIC), *AtTHI1* (THI1), both *AtTHIC* and *AtTHI1* (TT) or *AtTHIC*, *AtTHI1* and *AtTH1* (TTT), described by (Strobbe et al., 2021b), were utilized. To obtain an estimate of thiazole moiety accumulation in genetically engineered Arabidopsis plants, results from yeast assays using *thi4* and *thi6* were compared. Differences in results between both yeast assays (*thi4*-*thi6*) could be attributed to the accumulation of HET(-P), the thiazole intermediate in thiamin biosynthesis. Mean values ± SE of 3 (transgenic) or 6 (WT) biological replicates are shown. **(a)** Levels of total thiazole, as measured by LC-MS/MS after phosphatase treatment, are presented. **(b)** Differences in MTE results from *thi4* and *thi6* yeast assays are depicted, serving as an estimate for thiazole (HET-(P)) accumulation. The datasets (in A and B) were tested for normality using the Shapiro-Wilk test. In case of normality, statistically significant differences were detected via a two-sided T-test (scedasticity

depending on the outcome of a preceding F-test). In case of non-normality, Mann-Whitney U test was used to identify significant differences. Significant differences are indicated by a single asterisk ($p < 0.05$) or double asterisks ($p < 0.01$). For the engineered lines (B), the significant difference depict comparison with the wild type (WT).

Fig. S11

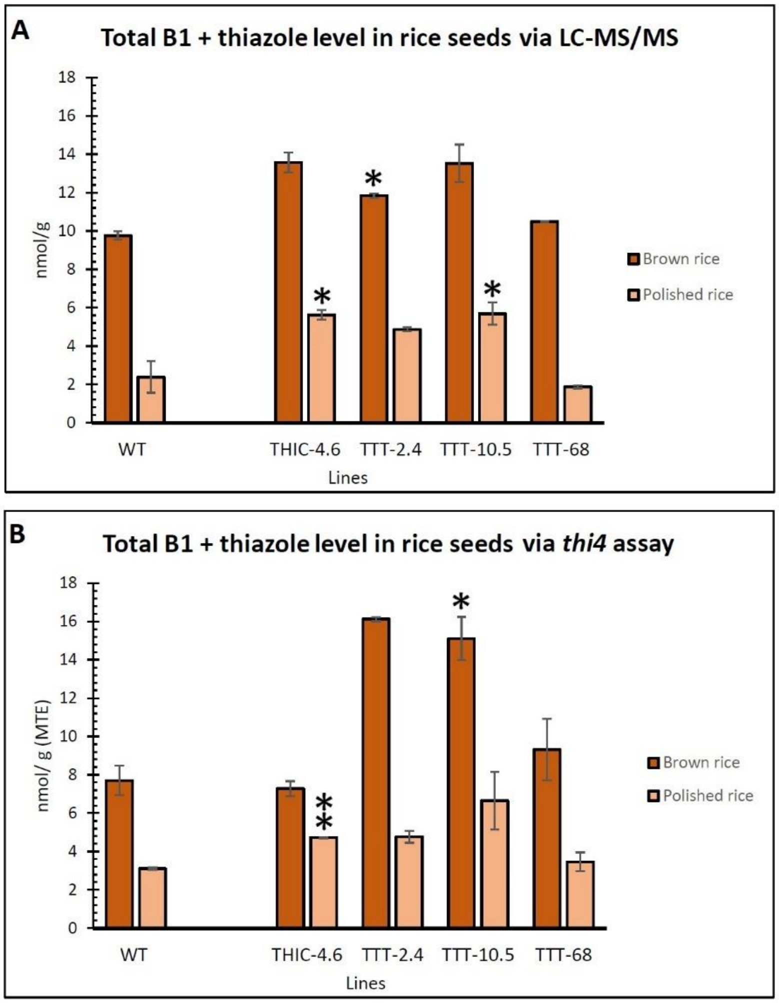

<u>Supporting Fig. S11. Preliminary results of utilizing *thi4* yeast assay to estimate sum of total vitB1and thiazole content in brown and polished rice seeds.</u> Genetically engineered rice lines, originating from a thiamin biofortification study (Strobbe et al., 2021a), were assessed via yeast assay and compared to LC-MS/MS methodology. **(a)** LC-MS/MS analysis of total B1 + thiazole content of both brown (unpolished) as polished rice seeds. **(b)** Total B1 + thiazole as estimated by *thi4* yeast assay. The yeast assay is able to detect differences between WT and engineered lines, both in polished and unpolished rice seed samples. Bars indicate mean ± SE of two repeats. Significant differences as compared to wild type (WT), as identified using a two-sided heteroscedastic T-test, are indicated by a single asterisk ($p < 0.05$) or double asterisks ($p < 0.01$).

Table S1 Supporting Table 1. Overview of the different yeast lines. Different *Saccharomyces cerevisiae strains* used in this study, are indicated. A list of growth restoring metabolites is provided, which is based on the genetic and biosynthetic knowledge of the specific mutant line. The source of which the specific strain was obtained is shown. Studies characterizing the mutated yeast genes are cited. The genetic background in which the mutant lines were created, are presented. It is important to note that the different lines contain additional auxotrophies (amino acids and uracil), which do not need to be taken into consideration, as all lines are grown on media supplemented with amino acids and uracil, provided by the Complete Supplement Mixture (Formedium, DCS0011).

Strain	Growth restoring metabolites	mutated genes(s)	Source	Reference	Background
RWY16	TPP, thiamin, TMP, HMP-PP, HMP-P, HMP	*THI5/THI11/THI12/THI13* (YFL058W/YJR156C/YNL332W/YDL244W)	Kindly donated by Dr. Raymond Wightman	(Wightman and Meacock, 2003)	BY4705 (MATα)
THI20/21	TPP, thiamin, TMP, HMP-PP	*THI20/21* (YOL055C/ YPL258C)	Produced by the team, using the published strategy	(Llorente et al., 1999;Kawasaki et al., 2005)	BY4741 (MATa)
THI4	TPP, thiamin, TMP, HET-P, HET	*THI4* (YGR144W)	Purchased from *DHARMACON* collection	(Chatterjee et al., 2011)	Homozygous diploid (BY4743, BY4741/BY4742)
THI6	TPP, thiamin, TMP	*THI6* (YPL214C)	Purchased from *EUROSCARF* collection	(Nosaka et al., 1994)	BY4741 (MATa)
THI80	TPP	*THI80* (YOR143C)	Purchased from *EUROSCARF* collection	(Nosaka et al., 1993)	Homozygous diploid (BY4743, BY4741/BY4742)

Table S2 Supporting Table 2. Identification of spiked metabolite in plant samples by using a scoring grid table. 60 different plant sample were subjected to the four yeast assays (RWY16, *thi4*, *thi6* and *thi20/21*) and were scored for each separate assay, the results of which are shown in Fig. S5. This allowed allocation of each sample to the baseline group or high metabolite group, which is indicated in the table as low and high, respectively. Attributing one sample to the 'low' or 'high' group, was performed for each of the four yeast assays. This allowed, based on the known array of metabolites rescuing the specific mutant strain (Fig. 1c), to score the samples based on the expected outcome (grid score) of a certain metabolite (Fig. 3b). For instance, a sample spiked with HMP is expected to fall into the 'high' group using the RWY16 assay, while being part of the 'low' or baseline group in the *thi4*, *thi6* and *thi20/21* assays (e.g. sample 1). This allowed, correct allocation of all spiked samples into the following groups (HMP spiked, HET spiked, B1 spiked and mock (non-spiked)).

Sample number	RWY16 Assay	thi4 Assay	thi6 Assay	thi20/21 Assay		Spiked metabolite
1	High	Low	Low	Low	→	HMP
2	Low	High	Low	Low	→	HET
3	High	High	High	High	→	B1
4	High	High	High	High	→	B1
5	High	Low	Low	Low	→	HMP
6	High	High	High	High	→	B1
7	Low	High	Low	Low	→	HET
8	High	High	High	High	→	B1
9	High	High	High	High	→	B1
10	Low	Low	Low	Low	→	Mock
11	High	High	High	High	→	B1
12	High	Low	Low	Low	→	HMP
13	Low	High	Low	Low	→	HET
14	High	High	High	High	→	B1
15	Low	Low	Low	Low	→	Mock
16	High	Low	Low	Low	→	HMP
17	High	High	High	High	→	B1
18	Low	Low	Low	Low	→	Mock
19	High	High	High	High	→	B1
20	Low	Low	Low	Low	→	Mock
21	High	High	High	High	→	B1
22	High	High	High	High	→	B1
23	High	Low	Low	Low	→	HMP
24	Low	High	Low	Low	→	HET
25	High	High	High	High	→	B1
26	High	High	High	High	→	B1
27	High	Low	Low	Low	→	HMP
28	High	High	High	High	→	B1
29	High	High	High	High	→	B1
30	Low	Low	Low	Low	→	Mock

**31**	Low	Low	Low	Low	→	Mock
**32**	High	High	High	High	→	B1
**33**	High	High	High	High	→	B1
**34**	High	Low	Low	Low	→	HMP
**35**	Low	High	Low	Low	→	HET
**36**	High	High	High	High	→	B1
**37**	Low	High	Low	Low	→	HET
**38**	High	Low	Low	Low	→	HMP
**39**	High	High	High	High	→	B1
**40**	High	High	High	High	→	B1
**41**	Low	Low	Low	Low	→	Mock
**42**	High	High	High	High	→	B1
**43**	Low	Low	Low	Low	→	Mock
**44**	High	High	High	High	→	B1
**45**	High	Low	Low	Low	→	HMP
**46**	Low	High	Low	Low	→	HET
**47**	High	High	High	High	→	B1
**48**	Low	Low	Low	Low	→	Mock
**49**	High	Low	Low	Low	→	HMP
**50**	Low	Low	Low	Low	→	Mock
**51**	Low	High	Low	Low	→	HET
**52**	High	High	High	High	→	B1
**53**	High	High	High	High	→	B1
**54**	Low	High	Low	Low	→	HET
**55**	High	High	High	High	→	B1
**56**	High	High	High	High	→	B1
**57**	High	High	High	High	→	B1
**58**	Low	High	Low	Low	→	HET
**59**	High	High	High	High	→	B1
**60**	High	High	High	High	→	B1

Methods S1 Yeast strains A relevant overview of *Saccharomyces cerevisiae* thiamin biosynthesis, as well as the utilized strains, is shown in Fig. 1. The applied microbiological assays were adapted from the originally described yeast assay using the *Saccharomyces cerevisiae* thiazole biosynthesis mutant, *thi4* (Kall, 2003;Raschke et al., 2007;Chatterjee et al., 2011;Mangel et al., 2017). Additionally, assays utilizing thiamin auxotrophic yeasts, mutated in the pyrimidine branch of thiamin biosynthesis, were included. These employ the strains RWY16 (Wightman and Meacock, 2003) and *thi20/21* (Llorente et al., 1999;Kawasaki et al., 2005), which are unable to synthesize thiamin due to defective HMP synthesis and HMP-P phosphorylation, respectively. For this work, the mutant *thi20/21* was obtained following the indications in the original publications (Llorente et al., 1999;Kawasaki et al., 2005), while the RWY16 mutant strain was kindly provided by Dr. R. Wightman. Moreover, *Saccharomyces cerevisiae* strains *thi6* (Nosaka et al., 1994) and *thi80* (Nosaka et al., 1993) were included. The former is unable to condense the pyrimidine and thiazole moieties to form TMP, while the latter is impaired in the synthesis of TPP. As these involve mutations further downstream in thiamin biosynthesis, these strains are hypothesized to allow specificity towards B1 vitamers when used in assays, limiting interference from intermediates.

Methods S2 Sample preparation Plant samples consisted of approximately 120 mg of Arabidopsis material (true leaves or complete seedlings; see above), with multiple samples representing biological replicates. In case of rice samples (Fig. S11), 100 mg (brown, unpolished rice) and 200 mg of polished rice was used. Polishing of rice samples consisted of overnight shaking on sandpaper (P180), resulting in approximately 10% seed weight loss (degree of

polishing) (Strobbe et al., 2021a). Upon collection, plant tissue samples were individually weighed, flash frozen in liquid nitrogen and subsequently stored at -80°C until further processing. The protocol for plant extract preparation was adapted from (Kall, 2003;Raschke et al., 2007;Mangel et al., 2017). Samples were pulverized and homogenized using a Retsch milling machine. A subsequent extraction and sterilization step was performed by addition of 1.5 ml of 22 mM sulfuric acid and incubating the samples at 95°C for 60 min (shaking at 500 rpm). The following steps (including the preparation of the assay) were performed in sterile conditions. Neutralization of the samples was achieved by addition of 240 µL sterile 3 M sodium acetate, ensuring a pH of 5.7. Excess of plant material (cell debris) was removed by centrifugation (5 min at 14000 *g* and 4°C). Subsequently, the clear supernatant was transferred to a new (sterile) tube. The final plant extracts were stored at -80°C until usage in the yeast assays (see further).

Methods S3 VitB1 standards An aqueous dilution series of sterile thiamin was made, which served as a standard to obtain a dose-response curve. This series consisted of 10 dilutions of thiamin, at a final concentration of 0 / 12.5 / 25 / 37.5 / 62.5 / 87.5 / 125 / 187.5 / 250 / 500 ng/mL, with a molar equivalent of 0 / 47 / 94 / 141 / 235 / 329 / 471 / 707 / 942 / 1885 nM thiamin. The latter is more relevant as the yeast assay is presumed to correlate to a molar concentration of different metabolites, referred to as molar thiamin equivalent (MTE).

Methods S4 Yeast cultures The different yeast strains were propagated on yeast extract-peptone-dextrose (YPD) medium, which allows growth of the auxotrophic mutant lines (Raschke et al., 2007;Mangel et al., 2017). A single colony growing on solid YPD medium (15 g/L agar, Sigma-Aldrich) was selected per each strain and sub-cultured on liquid thiamin free yeast medium

(TFYM) supplemented with 100 ng/mL thiamin. TFYM comprises yeast nitrogen base without amino acids and without thiamin (CYN4701, ForMedium), enriched with complete supplement medium (DCS0011, ForMedium) and 1% sucrose (following the manufacturer's recommended concentrations). In practice, 6.9 g CYN4701, 790 mg DCS0011 and 10 g sucrose are dissolved in 500 ml distilled water and autoclaved to yield double concentrated TFYM (2x TFYM). The yeast strains were grown for 1 day at 28°C shaking at 200 rpm, until an optical density (OD) above 1 was reached. Subsequently, the cultures were pelleted (1700 *g*, 5 min) and resuspended in 0.85% NaCl, which was repeated 3 times to eliminate any residual presence of thiamin. The yeast cells were again pelleted (1 700 *g*, 5 min) and resuspended in TFYM (no vitB1) to reach an OD of 0.5. The yeast cultures were stored at 4°C until usage in the microbiological assays.

Methods S5 Assay protocol, conditions and data acquisition The yeast assays were performed using 24-well plates (CELLSTAR, Greiner Bio-One), in sterile conditions. The following constituents were added to each well: 250 µL of 2xTFYM (double concentrated TFYM), 50 µL yeast (OD 0.5), 233 µL water, 67 µL sample (plant extract or standard). Each standard concentration was analyzed in quadruplicate. The 24-well plates, harboring 600 µL assay volume in each well, were incubated at 28°C for 17 hours (200 rpm). The optical density of the wells was measured using a TECAN Infinite 200 Pro plate reader, which allows rapid acquisition of the OD data. The OD data of the thiamin standards (4 of each concentration) were utilized to obtain a dose-response curve. For all analyzed yeast strains, a dose-response curve was constructed, which was used to determine the thiamin level (or molar thiamin equivalent, MTE) present in the plant extracts. The amount of metabolites measured was calculated from the MTE present in the plant extracts, taking the initial weight of the plant sample into consideration (± 120 mg).

Methods S6 Spiking of plant extracts A schematic overview of the experimental setup is depicted in Fig. S3. To minimize biological variation, all samples were derived from a pooled set of plant extracts. The pool was divided into individual samples and spiked, blind-coded for the analyzing researcher, with a molar amount of metabolites (0.2 µM HMP, HET, thiamin, TMP or TPP), two times exceeding the molar thiamin equivalent measured in non-spiked samples using the *thi6* assay (0.1 µM). By doing so, the final spiked samples would exceed the MTE of non-spiked samples by 3-fold. Before spiking, each sample consisted of 1.8 mL homogenized plant extract (containing 0.1 µM MTE). These samples were spiked with 200 µL of 2 µM metabolite (HMP, HET, thiamin, TMP or TPP), in buffer solution (1.5 ml of 22 mM sulfuric acid, neutralized to pH 5.7) with 240 µL sterile 3 M sodium acetate), thereby intended to reach a MTE of 0.3 µM in the 2.0 mL samples. Another set of samples was supplemented with only sterile extraction buffer, referred to as ‘mock’. The sample sets were randomized and assessed using the described yeast assays.

Supporting References

Chatterjee, A., Abeydeera, N.D., Bale, S., Pai, P.J., Dorrestein, P.C., Russell, D.H., Ealick, S.E., and Begley, T.P. (2011). Saccharomyces cerevisiae THI4p is a suicide thiamine thiazole synthase. *Nature* 478**,** 542-U146.

Kall, M.A. (2003). Determination of total vitamin B-6 in foods by isocratic HPLC: a comparison with microbiological analysis. *Food Chemistry* 82**,** 315-327.

Kawasaki, Y., Onozuka, M., Mizote, T., and Nosaka, K. (2005). Biosynthesis of hydroxymethylpyrimidine pyrophosphate in *Saccharomyces cerevisiae. Current Genetics* 47**,** 156-162.

Llorente, B., Fairhead, C., and Dujon, B. (1999). Genetic redundancy and gene fusion in the genome of the baker's yeast Saccharomyces cerevisiae: functional characterization of a three-member gene family involved in the thiamine biosynthetic pathway. *Molecular Microbiology* 32**,** 1140-1152.

Mangel, N., Fudge, J.B., Fitzpatrick, T.B., Gruissem, W., and Vanderschuren, H. (2017). Vitamin B-1 diversity and characterization of biosynthesis genes in cassava. *Journal of Experimental Botany* 68**,** 3351-3363.

Nosaka, K., Kaneko, Y., Nishimura, H., and Iwashima, A. (1993). Isolation and characterization of a thiamin pyrophosphokinase gene, *THI80*, from *Saccharomyces Cerevisiae*. *Journal of Biological Chemistry* 268**,** 17440-17447.

Nosaka, K., Nishimura, H., Kawasaki, Y., Tsujihara, T., and Iwashima, A. (1994). Isolation and characterization of the *THI6* gene encoding a bifunctional thiamin-phosphate pyrophosphorylase hydroxyethylthiazole kinase from *Saccharomyces cerevisiae*. *Journal of Biological Chemistry* 269**,** 30510-30516.

Raschke, M., Burkle, L., Muller, N., Nunes-Nesi, A., Fernie, A.R., Arigoni, D., Amrhein, N., and Fitzpatrick, T.B. (2007). Vitamin B1 biosynthesis in plants requires the essential iron-sulfur cluster protein, THIC. *Proceedings of the National Academy of Sciences of the United States of America* 104**,** 19637-19642.

Strobbe, S., Verstraete, J., Stove, C., and Van Der Straeten, D. (2021a). Metabolic engineering of rice endosperm towards higher vitamin B1 accumulation. *Plant Biotechnology Journal* 19, 1253-1267.

Strobbe, S., Verstraete, J., Stove, C., and Van Der Straeten, D. (2021b). Metabolic engineering provides insight into the regulation of thiamin biosynthesis in plants. *Plant Physiology* 186, 1832-1847.

Verstraete, J., Strobbe, S., Van Der Straeten, D., and Stove, C. (2020). The first comprehensive LC–MS/MS method allowing dissection of the thiamine pathway in plants. *Analytical Chemistry* 92**,** 4073-4081.

Wightman, R., and Meacock, P.A. (2003). The *THI5* gene family of Saccharomyces cerevisiae: distribution of homologues among the hemiascomycetes and functional redundancy in the aerobic biosynthesis of thiamin from pyridoxine. *Microbiology* 149**,** 1447-1460.
